# MIST1 regulates endoplasmic reticulum stress-induced hepatic apoptosis as a candidate marker of fatty liver disease progression

**DOI:** 10.1038/s41419-024-07217-0

**Published:** 2024-11-08

**Authors:** Sumin Hur, Haengdueng Jeong, Keunyoung Kim, Kwang H. Kim, Sung Hee Kim, Yura Lee, Ki Taek Nam

**Affiliations:** 1 Severance Biomedical Science Institute, Graduate School of Medical Science, Brain Korea 21 Project, Yonsei University College of Medicine, Seoul, Korea; 2 Department of Pharmacy, Kangwon National University College of Pharmacy, Chuncheon, Korea

**Keywords:** Apoptosis, Non-alcoholic fatty liver disease

## Abstract

The liver regenerates after injury; however, prolonged injury can lead to chronic inflammation, fatty liver disease, fibrosis, and cancer. The mechanism involving the complex pathogenesis of the progression of liver injury to chronic liver disease remains unclear. In this study, we investigated the dynamics of gene expression associated with the progression of liver disease. We analyzed changes in gene expression over time in a mouse model of carbon tetrachloride (CCl_4_)-induced fibrosis using high-throughput RNA sequencing. Prolonged CCl_4_-induced liver injury increased the expression levels of genes associated with the unfolded protein response (UPR), which correlated with the duration of injury, with substantial, progressive upregulation of muscle, intestine, and stomach expression 1 (Mist1, bhlha15) in the mouse fibrosis model and other liver-damaged tissues. Knockdown of *MIST1* in HepG2 cells decreased tribbles pseudokinase 3 (TRIB3) levels and increased apoptosis, consistent with the patterns detected in *Mist1*-knockout mice. MIST1 expression was confirmed in liver tissues from patients with metabolic dysfunction-associated steatohepatitis and alcoholic steatohepatitis (MASH) and correlated with disease progression. In conclusion, MIST1 is expressed in hepatocytes in response to damage, suggesting a new indicator of liver disease progression. Our results suggest that MIST1 plays a key role in the regulation of apoptosis and TRIB3 expression contributing to progressive liver disease after injury.

## Introduction

The functional unit of the liver is the lobule, which has a hexagonal structure centered on a single central vein surrounded by six portal triads. Hepatocytes are epithelial cells that exhibit a cord-like arrangement in a row centered on the central vein, playing a key role in metabolizing drugs, ethanol, and fatty acids, and also serving as fat storage centers. Peri-central and peri-portal hepatocytes express different sets of genes, and a gradient of gene expression levels is formed across the hepatocytes located between these two zones [1]. The pathogenesis of chronic liver disease is regulated by interactions among various cell types, genes, and metabolic pathways. However, the progression of chronic liver disease cannot be attributed to a single mechanism [2]. Therefore, genetic profiling over the course of the progression of liver injury to chronic liver disease is crucial in understanding this process.

Exposure to the continuous endoplasmic reticulum (ER) stress leading to an unfolded protein response (UPR) has emerged as an important mechanism contributing to pathological conditions of the liver such as hepatic steatosis, inflammation, and cell death [3, 4]. This type of chronic injury is critical for the development of steatosis and the progression of metabolic-associated fatty liver disease (MAFLD) in humans [4–6]. The UPR is a component of the integrated stress response, which is activated in response to ER stress and changes in lipid synthesis and protein expression. The UPR is categorized into adaptive UPR and terminal UPR depending on the degree and duration of ER stress [3]. The adaptive UPR caused by mild or acute ER stress helps to maintain protein homeostasis, and lipid synthesis, and enhances cell survival in the face of ER stress. The genetic mechanisms and pathways regulating the UPR are coming to light, suggesting potential targets for chronic liver disease. Inositol-requiring enzyme-1 (IRE1) is an inducer of the UPR, which splices x-box binding protein 1 (XBP1) mRNA, consequently inducing the expression of genes related to ER-associated degradation, chaperone proteins, and insulin-dependent de novo lipogenesis, ultimately resulting in stress adaptation [7–11]. Specifically, XBP1 promotes the expression of MIST1 during cell differentiation or under ER stress [12, 13]. MIST1 serves as a transcription factor that regulates the organization of secretory cell structures in gastrointestinal cells, acinar cells of the pancreas, and salivary glands [14–19]. MIST1 is also involved in cell reprogramming. Previous reports have suggested that the overexpression of MIST1 in parietal cells of the stomach results in increased cell polarity and an enlarged ER [14, 20]. MIST1 expression was reported to disappear in a condition of pancreatic acinar-to-ductal metaplasia or spasmolytic polypeptide-expressing metaplasia [21]. However, the role of MIST1 in adult hepatocytes remains obscure, as MIST1 is rarely expressed under homeostatic conditions [20, 22].

Accordingly, the aim of this study was to investigate changes in gene expression dynamics associated with chronic liver damage induced under continuous ER stress and to identify genes that reflect disease progression. The intraperitoneal injection of carbon tetrachloride (CCl_4_) induces continuous ER stress and is commonly used as a rodent model of liver damage, which is particularly evident in the peri-central hepatocytes [23, 24]. Therefore, we performed sequential transcriptomics using liver specimens collected during long-term CCl_4_ injury in mice and in tunicamycin-treated human HepG2 cells. Our transcriptome data demonstrated that *Mist1* expression is substantially induced in damaged hepatocytes and contributes to ER-induced apoptosis. Therefore, we further focused on the roles of MIST1 in the progression of chronic liver injury to chronic liver disease using *Mist1*-knockout (*Mist1*^KO^) mice and *MIST1* small interfering RNA (siRNA)-transfected HepG2 cells. Collectively, we expect the results to provide new insight into the gene expression changes induced under chronic ER stress contributing to liver injury and progression, and identify new targets for diagnosis, disease monitoring, and potential therapeutics.

## Results

### Long-term CCl_4_ treatment increased the expression of genes related to ER stress and apoptosis

**Fig. 1:**
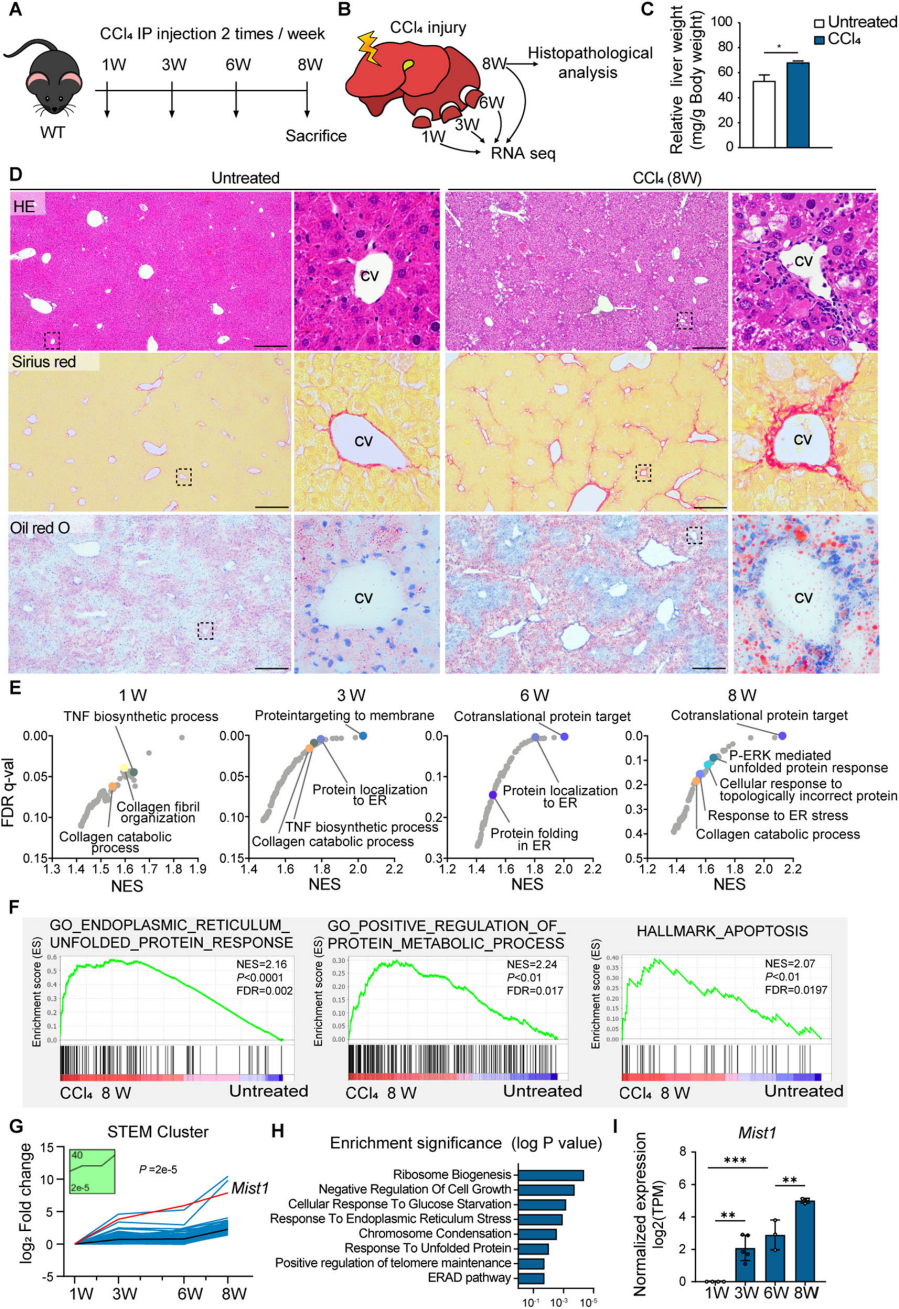
Long-term CCl_4_ treatment increased the expression of genes related to ER stress and apoptosis. **A** Schematic of the experimental design. **B** Liver biopsy collection. **C** Relative liver weight (g) to body weight (100 mg) at 8 weeks (*n* = 3 per each group). *p < 0.05; data are presented as a mean ± SEM. **D** HE, Sirius red, and Oil red O staining of control and CCl_4_-treated livers. Scale bar, 400 μm (cv central vein). **E** Bubble plots of false discovery rate (FDR) *q*-value vs normalized expression score (NES) for each gene set (dot). **F** Gene distribution for gene sets related to ER stress, protein metabolism, and apoptosis. *p*, nominal *p*-value. **G** Trajectory plot of cluster 40 identified by short time-series expression miner (STEM) analysis (red line = *Mist1* expression). **H** Top eight significantly enriched Gene Ontology terms of cluster 40. **I** *Mist1* mRNA expression level over time during CCl_4_ administration (TPM transcript per million; *n* ≥ 3).

Liver biopsy specimens were obtained from the medial lobe of the liver for bulk RNA sequencing at 1, 3, 6, and 8 weeks in the same mice after the initial injection of CCl_4_ (Figs. 1A, B and S1A). Long-term CCl_4_ treatment caused an increase in liver mass and liver injury characterized by fibrosis and steatosis (Figs. 1C, D and S2A–B). At 8 weeks, there was hepatocyte ballooning encircling the compromised central vein (Fig. 1D) and central–central bridging fibrosis that formed a pseudo-lobular structure accompanied by accumulation of lipid droplets (Figs. 1D and S2C). Notably, apoptotic cells were observed around the fibrotic septum, and their proportion increased from weeks 1 to 8 (Fig. S2D, E).

Gene sets associated with wound-healing processes were enriched in CCl_4_-treated mice at 1 week, including tumor necrosis factor synthesis associated with the inflammatory phase and collagen synthesis associated with the proliferative phase, compared to those of vehicle-treated mice (Fig. 1E). At 3 weeks, gene sets associated with early wound healing remained altered, whereas from 6 weeks onward, gene sets related to translational and post-transcriptional processes were most significantly enriched (Fig. 1E). Supporting the histopathological findings, genes related to ER stress, the UPR, and apoptosis were enriched at 8 weeks of CCl_4_ treatment (Fig. 1E, F).

### *Mist1* expression is increased in hepatocytes of the injured liver

**Fig. 2:**
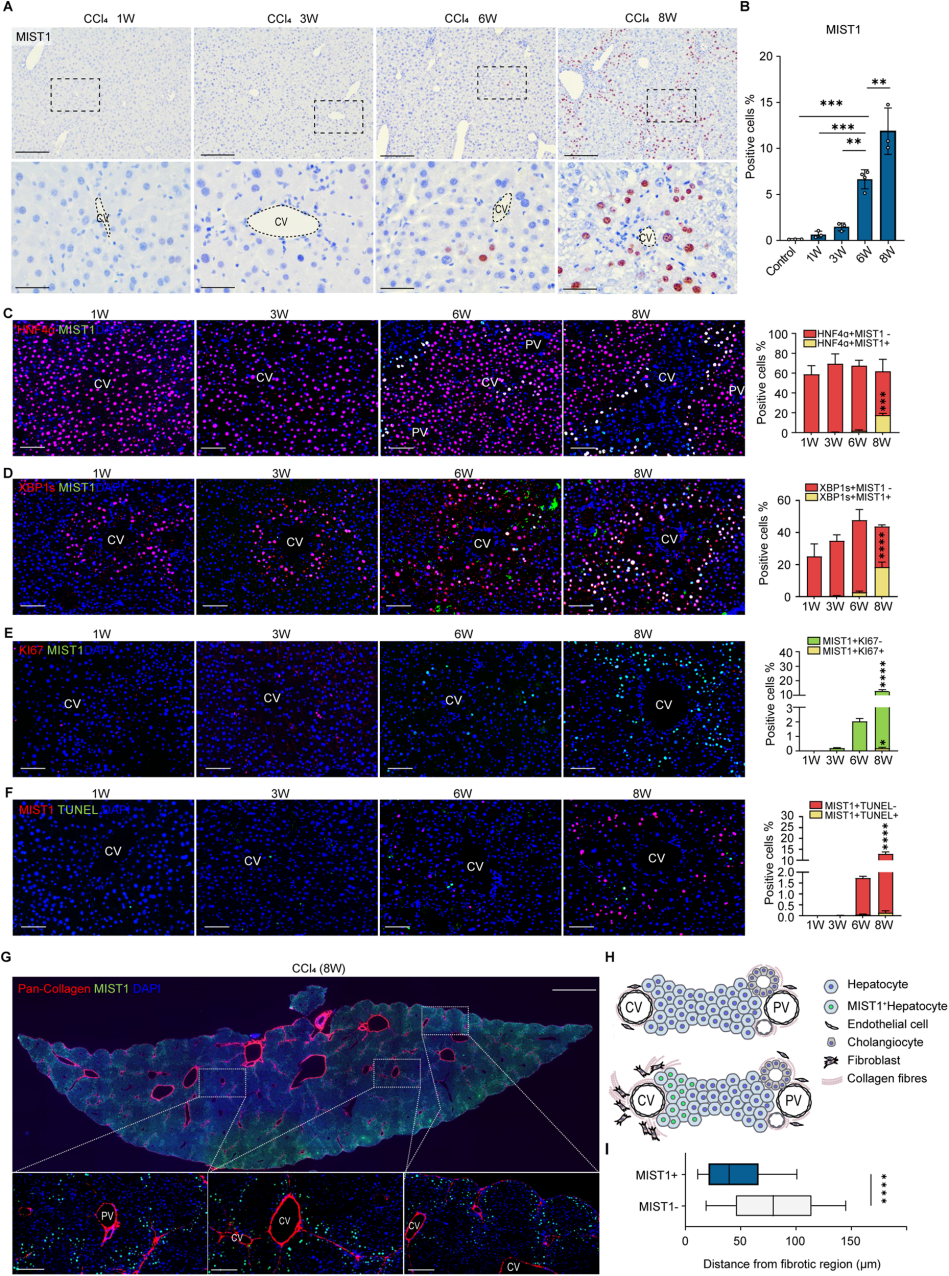
Expression of MIST1 is increased in hepatocytes of CCl_4_-treated mice. **A** Immunohistochemistry of MIST1 expression over time during CCl_4_ administration. (Scale bar, 400 μm). **B** Percentile of DAB-positive nuclei in whole-tissue slide sections (*n* = 3; control, no treatment; W week). **C**–**G** Immunofluorescence for MIST and **C** HNF4α, **D** XBP1-s, **E** KI67, **F** TUNEL, and **G** collagen. Scale bars: 400 μm (**C**–**F**), 100 μm (**D**), 2 mm and 200 μm (**G**), (CV central vein, PV portal vein). **H** MIST1-positive cells among whole cell lineages in the CCl_4_-treated liver. **I** Distance distributions of MIST1-negative cells (*n* = 28 074) and MIST1-positive cells (*n* = 9918) from collagen-positive regions. ****p < 0.0001 (two-tailed unpaired Student’s *t*-test). All data are presented as mean ± SEM.

Because of the complexity of gene expression during injury progression, we performed pattern analysis using a short time-series expression miner on the RNA-sequencing data from the liver lobes of mice treated with CCl_4_ for 1–8 weeks, which identified a collection of 16 clusters organized based on similarities in expression patterns. In the time-series data (Fig. S2F), a pattern of initial increase followed by a decrease was identified in cluster 43, and these genes were associated with collagen-related Gene Ontology (GO) terms (Fig. S2G, H). Cluster 47 was characterized by increased expression levels from weeks 3 to 6, followed by a return to basal levels, including genes related to mitochondrial respiration (Fig. S2I, J). The genes in cluster 12, which displayed a sustained decrease throughout the experimental period, had a significant presence of Rho and GTPase GO terms and were also associated with epithelial cell apoptosis GO terms (Fig. S2K, L). Among these clusters, we specifically focused on cluster 40, which exhibited a monotonically increasing pattern, similar to the histopathological pattern (Fig. 1G). GO analysis of cluster 40 revealed significant enrichment of gene sets related to protein translation, the UPR, and ER stress, as well as enrichment of genes related to glucose starvation and chromosome condensation (Fig. 1H). Among these genes, *Mist1* showed a distinct fold change (Fig. 1G, I). Moreover, immunohistochemistry images showed that the MIST1 expression level gradually increased around the CCl_4_-damaged central vein and along the fibrotic septum (Fig. 2A, B), and was predominantly expressed in HNF4α-positive hepatocytes at 8 weeks (Fig. 2C). Notably, the key transcription factor of the UPR, XBP1-s (Figs. 2D and S3J), was also prominently expressed at 6–8 weeks after CCl_4_ injury with approximately one in three XBP1-s-positive cells also expressing MIST1 at week 8.

As the UPR and ER stress are associated with cell survival during injury, we further investigated the putative role of MIST1 in cell proliferation and apoptosis. The ratio of MIST1-only positive nuclei to the total number of DAPI-stained nuclei was 2.02% ± 0.35% and 13.9% ± 0.4%, respectively, at weeks 6 and 8. However, KI67 and MIST1 double-positive cells accounted for only 0 to 0.14% ± 0.1% of total DAPI-stained nuclei; therefore, a significant proportion of MIST1-positive cells displayed minimal signs of active proliferation (Fig. 2E). The number of TUNEL-positive nuclei showed an increasing trend over time, similar to the pattern detected for MIST-1-positive nuclei (Figs. 2A and S2E). However, among the total DAPI-positive nuclei, only 0.18% ± 1% were labeled for TUNEL and simultaneously expressed MIST1 at 8 weeks (Fig. 2F). These results suggested that MIST1 is primarily expressed in cells under ER stress and not in proliferating and apoptotic phases.

### Induction of MIST1 is recapitulated in damaged sites of diverse injury models

Because CCl_4_ administration caused pericentral vein injuries accompanied by the accumulation of collagen fibers, we sought to identify the precise location of MIST1 induction in the liver after injury (Fig. 2G). Whole-tissue scanning slides stained with pan-collagen and MIST1 were used to calculate the distance between each MIST1-positive nucleus and collagen-positive region. Notably, MIST1 expression was prominent in the surrounding hepatocytes as the fibrotic region near the central vein expanded due to injury (Fig. 2G, I). Moreover, we observed different patterns of MIST1 expression in different liver damage models (Fig. S3A–I). The MIST1 expression level increased in mice subjected to bile duct ligation, which induces periportal damage, and the MIST1-expressing hepatocytes were located around the portal triad (Fig. S3A–C). In the MASH model induced by choline deficiency, L-amino acid deficiency, and a high-fat diet (CDAHFD), MIST1 expression was observed throughout the liver lobule, and its expression level was significantly increased compared to that in the group fed a regular diet (Fig. S3D–F). After treatment with the ER stress inducer tunicamycin, MIST1-positive cells were observed throughout the liver, which had substantially decreased by 24 h after CCl_4_ treatment, with only the periportal hepatocytes remaining MIST1-positive (Fig. S3G–I). In conclusion, MIST1 is specifically and transiently expressed in hepatocytes in the region where liver injury occurs.

### *Mist1*^KO^ mice are more susceptible to CCl_4_-induced stress

**Fig. 3:**
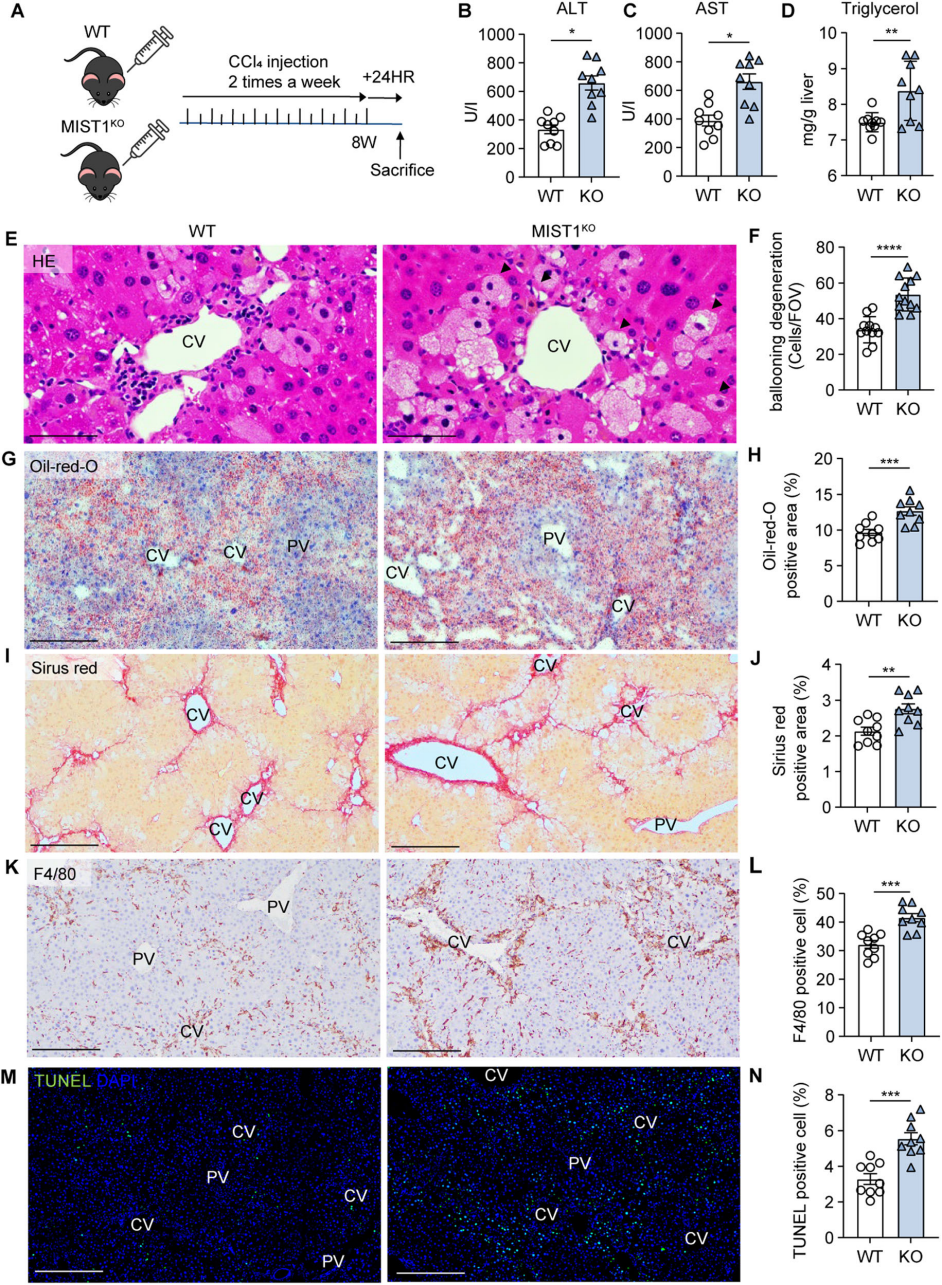
*Mist1*^KO^ mice are more susceptible to CCl_4_-induced stress. **A** Experimental scheme. **B**, **C** Levels of blood serum transaminases; *n* ≥ 3 per group. **D** Triglyceride content in the caudate lobe; *n* = 9 per group. **E**, **G**, **I**, **K**, and **M** H&E, Oil-red-O, Sirius red, F4/80 immunohistochemistry, and TUNEL immunofluorescence in *Mist1*^KO^ mice and wild-type (WT) littermates treated with CCl_4_. **F** Ballooning degeneration, **H** lipid droplet accumulation, **J** fibrotic area, **L** macrophage recruitment, and **N** apoptotic cell counts in tissue sections; *n* = 9 per group, (CV central vein, PV portal vein). All data are presented as mean ± SEM.

ALT and AST levels and the liver triglyceride content were significantly elevated in CCl_4_-treated *Mist1*^KO^ mice compared to control mice (Fig. 3A–D). More hepatocytes exhibiting ballooning degeneration across a larger area were detected in *Mist1*^KO^ mice than in WT mice after 8 weeks of CCl_4_ treatment (Fig. 3E, F). Furthermore, *Mist1*^KO^ mice exhibited severe damage in various categories, including lipid droplet accumulation and fibrosis (Fig. 3G–J). Immunohistochemistry revealed a higher distribution of macrophages in a broader area extending from the central vein and fibrotic septum in *Mist1*^KO^ than in WT mice (Fig. 3K, L). There were more TUNEL-labeled nuclei in the livers of *Mist1*^KO^ mice than in those of WT mice (Fig. 3M, N). However, in the homeostatic state, in which MIST1 expression was not induced in hepatocytes (Fig. 2A), there were no differences between WT and *Mist1*^KO^ mice in lipid accumulation, macrophage percentage, or percentage of TUNEL-labeled nuclei (Fig. S4A–F). These findings suggest that MIST1 plays a protective role against ER stress-induced injury and apoptosis in hepatocytes.

### *Mist1* knockout promotes apoptosis in mouse hepatocytes

**Fig. 4:**
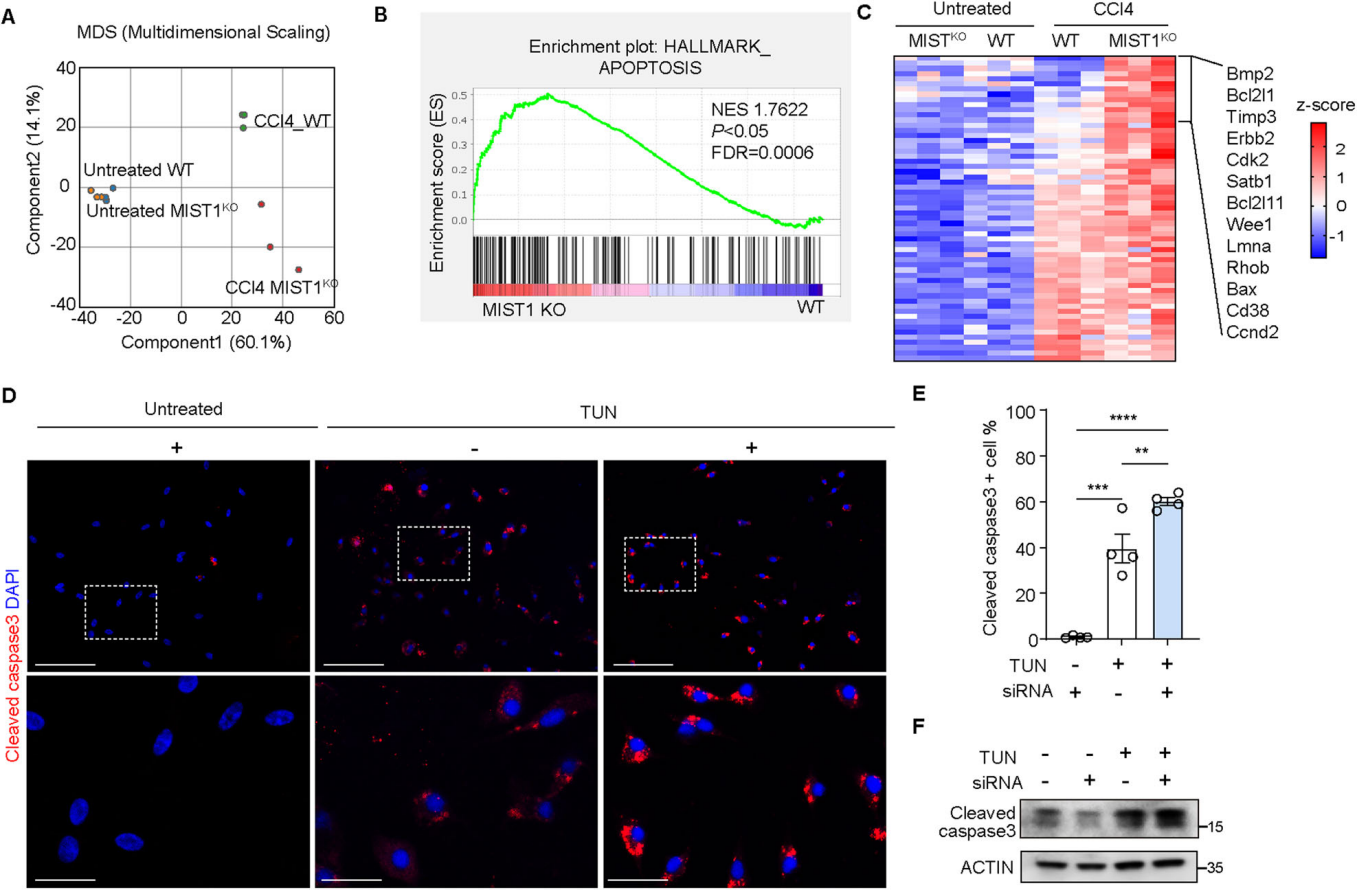
MIST1 knockout increased apoptosis in hepatocytes. **A** Multi-dimensional scaling plot of RNA-sequencing datasets from non-treated *Mist1*^KO^ mice (orange) and wild-type (WT) littermates (blue) and CCl_4_-treated *Mist1*^KO^ mice (red) and WT littermates (green). **B** Gene set enrichment analysis comparing CCl_4_-treated *Mist1*^KO^ and other groups at 8 weeks, (NES normalized expression score, *p* nominal *p*-value, FDR false discovery rate *q*-value). **C** Heatmap of gene set related to Hallmark of apoptosis. **D** Immunocytochemistry of cleaved caspase-3 in negative control (−) and *MIST1* siRNA (+)-treated HepG2 cells with (+) or without (−) tunicamycin supplementation. **E** Percentage of cleaved caspase 3-positive cells over time in HepG2 cells; *n* = 4. All data are presented as mean ± SEM. **p < 0.01, ***p < 0.001, ****p < 0.0001 (one-way ANOVA with Dunnett’s multiple comparison). **F** Western blot for cleaved caspase-3 in HepG2 cells cultured with (+) or without (−) tunicamycin, *MIST1* siRNA (+), and/or control siRNA (−). All data are presented as mean ± SEM.

Transcriptome analysis of liver sections demonstrated minor differences between untreated WT and *Mist1*^KO^ mice (Figs. 4A and S5A, B), whereas distinct variations in gene expression were observed between the two groups after CCl_4_ administration (Figs. 4A and S5A–C).

Gene set enrichment analysis revealed significant enrichment of genes related to the inflammatory response and apoptosis in CCl_4_-treated *Mist1*^KO^ mice compared to those in WT mice (Figs. 4B and S5D–F). Consistently, the heatmap indicated that transcripts associated with apoptosis (encoding BMP2 and BCL-2 family proteins) were significantly increased in CCl_4_-treated *Mist1*^KO^ mice compared with those in CCl_4_-treated WT mice (Fig. 4C). Immunoblotting showed that IRE1-XBP1, a canonical ER stress pathway upstream of MIST1, was upregulated in both WT and *Mist1*^KO^ mice in response to CCl_4_ administration, with no difference in expression between the two groups (Fig. S5G, H). Likewise, *Xbp1* transcript levels did not differ between the two groups (Fig. S5I). These results suggested that liver cells can respond to ER stress even under MIST1-deficient conditions. In contrast, the expression of CHOP, a transcription factor associated with the apoptosis pathway, was upregulated in CCl_4_-treated *Mist1*^KO^ mice compared to WT mice, accompanied by an increase in cleaved caspase 3 (Fig. S5G). Consistently, in vitro experiments showed that treatment with tunicamycin, an antibiotic that induces ER stress, increased the expression of cleaved caspase-3 in the cytoplasm of HepG2 cells (Fig. 4D, E), and *MIST1* knockdown aggravated this change (Fig. 4F).

### MIST1 is a transcription factor for TRIB3

**Fig. 5:**
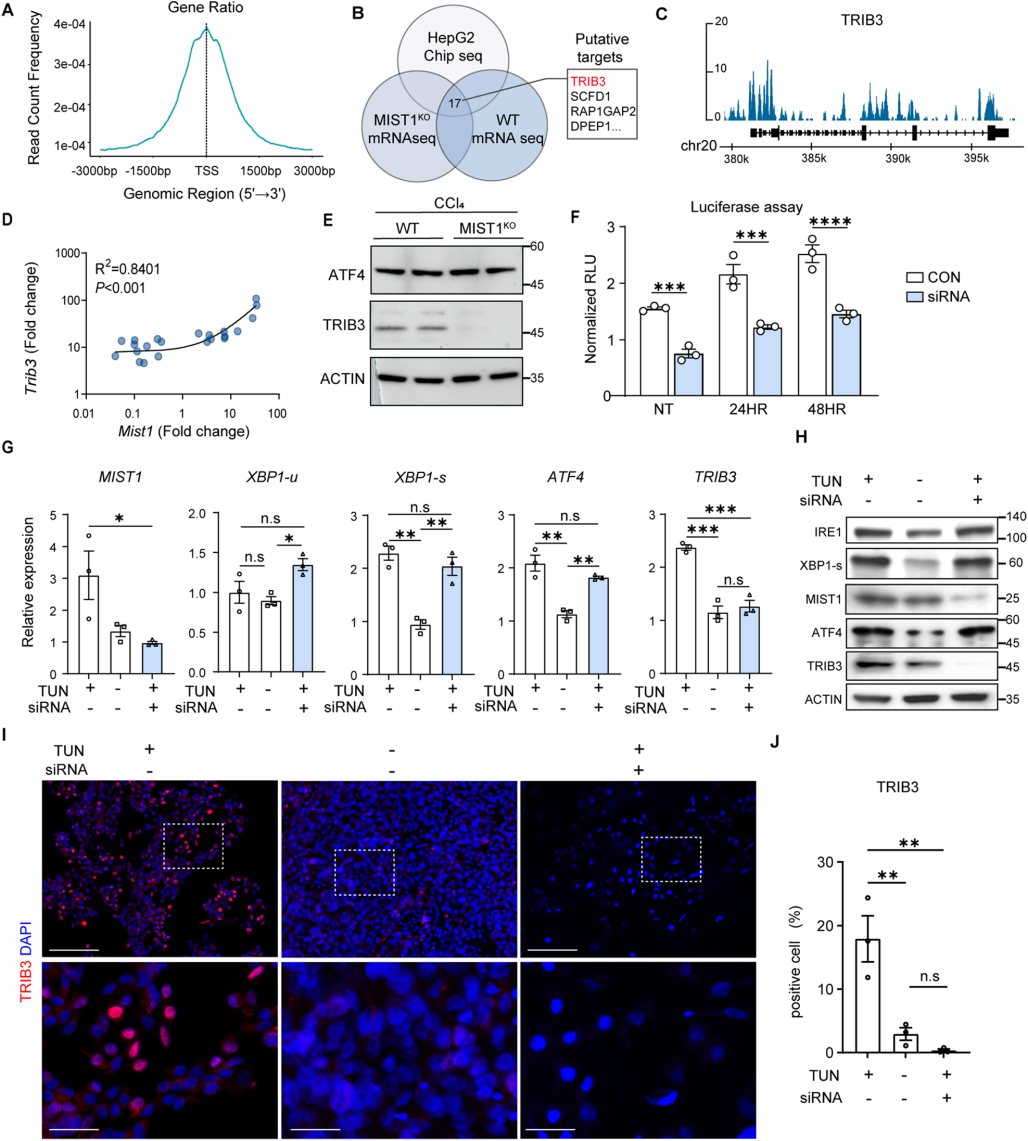
MIST1 is a putative transcription factor of *TRIB3.* **A** Distribution of MIST1 binding frequency around transcription start sites. **B** MIST1 target candidate genes were identified as an intersection of the MIST1-bound genes, MIST1-positive correlated genes, and genes with reduced expression in *Mist1*^KO^ mice. **C** ChIP-seq binding peaks of MIST1 around target genes. **D** Scatterplot of normalized expression of candidate MIST1 target genes of CCl_4_-treated and wild-type (WT) mice. **E** Western blot for TRIB3 and ATF4 in liver specimens from CCl_4_-treated control (WT) and *Mist1*^KO^ mice. **F** Relative luciferase activity of the *TRIB3* promoter with tunicamycin-induced ER stress in HepG2 cells with MIST1 knockdown and control cells; *n* = 3 per each group. **G**, **H** Relative mRNA and protein expression of ER stress-related molecules in HepG2 cells cultured with (+) or without (−) tunicamycin, *MIST1* siRNA, and/or control siRNA; *n* = 3 per each group. **I** Immunocytochemistry of TRIB3 in control and siRNA-treated HepG2 cells. **J** TRIB3-positive cells in tunicamycin-treated HepG2 cells over time; *n* = 3 per each group. All data are presented as mean ± SEM. *p < 0.05, **p < 0.01, ***p < 0.001, ****p < 0.0001 (unpaired Student’s *t*-test or one-way ANOVA with Dunnett’s multiple comparisons).

Since MIST1 is well known for its function as a transcription factor [25], we aimed to identify its putative target genes. ChIP-seq data from HepG2 cells identified 3664 candidate genes with MIST1 binding sites within 2500 bp of the transcription start site (Fig. 5A). Transcriptomics of liver tissues identified 218 putatively significantly downregulated genes in *Mist1*^KO^ mice compared to WT mice at 8 weeks after CCl_4_ treatment; among these, 17 genes showed a positive correlation with MIST1 in the CCl_4_-treated WT group (Figs. 5B, D and S6A–C), as was the case in the array data from MASH patients (Fig. S6D), with the highest predictive accuracy found for *Trib3* (Fig. 5B–D). However, there was no difference in the presence of the UPR regulator and TRIB3 inducer activating transcription factor 4 (ATF4) [26, 27]. This finding suggested that MIST1 may regulate TRIB3 through an ATF4-independent pathway under ER stress (Fig. 5E). Most importantly, the promoter activity of TRIB3 increased in response to tunicamycin treatment, and this effect was reduced by MIST1 knockdown (Fig. 5F). Consistent with this hypothesis, the mRNA expression levels of *XBP1s* and *ATF4* increased with tunicamycin treatment but were not affected by *MIST1* knockdown with siRNA (Fig. 5G, H). However, *MIST1* knockdown reduced TRIB3 expression at both the mRNA and protein levels (Fig. 5G, H). Immunocytochemistry showed that *MIST1* knockdown reduced the number of tunicamycin-treated cells expressing TRIB3 in the nuclei (Fig. 5I, J).

### MIST1 expression is correlated with human disease progression

**Fig. 6:**
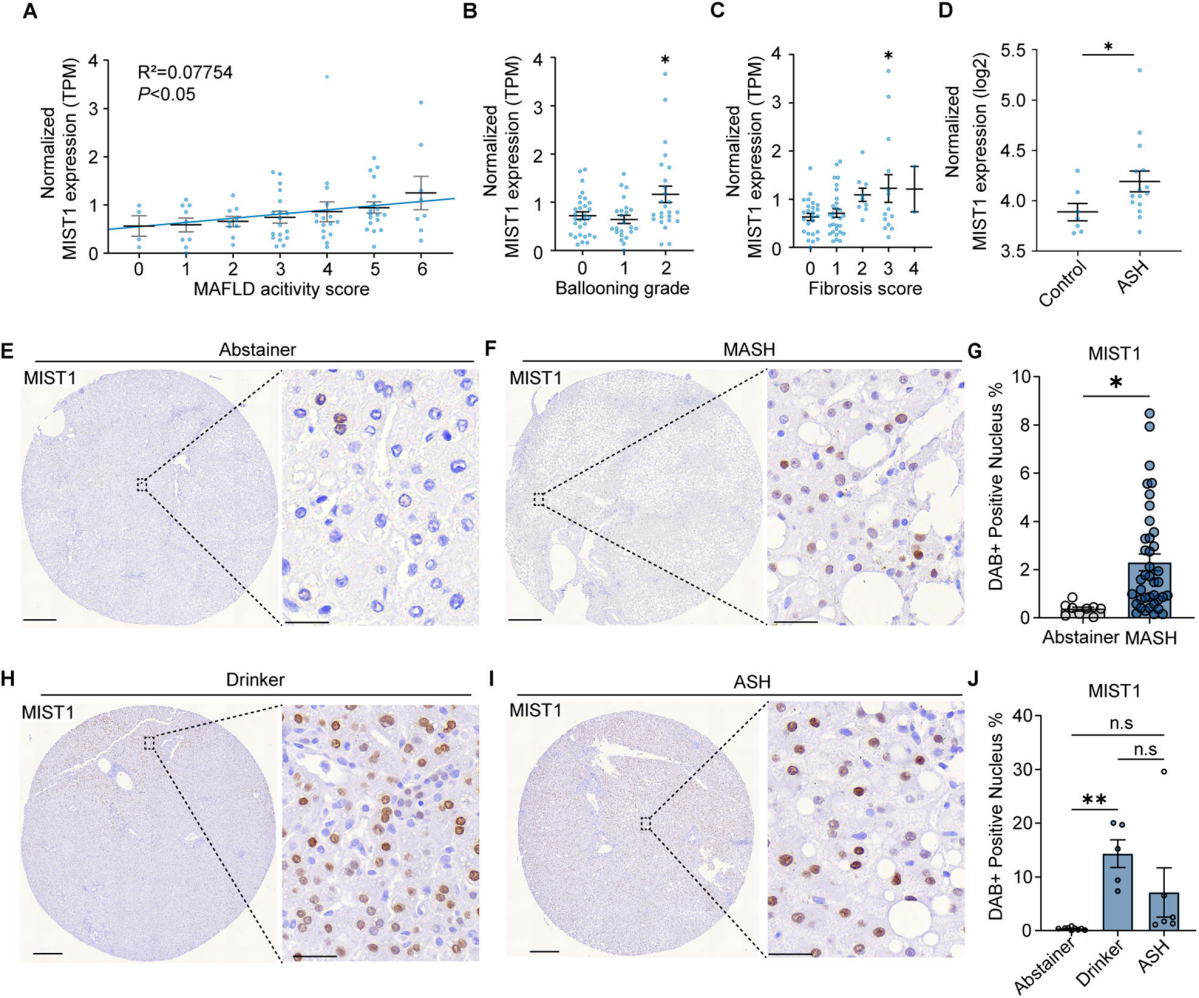
Expression of MIST1 is correlated with human disease progression. **A**–**C** Correlations (Pearson’s correlation coefficient) between *MIST1* expression and **A** MAFLD activity score, **B** ballooning grade, and **C** fibrosis score. **D** Relative MIST1 expression in ASH patients and healthy donors (GSE28619). **E**, **F** Immunohistochemistry for MIST1 in tissue microarrays from alcohol abstainers and patients with MASH (scale bar = 400 μm, 100 μm). **G** MIST1-positive cells in the tissue sections (*n* = 9 abstainer group, *n* = 39 MASH group). **H**, **I** Immunohistochemistry for MIST1 in tissue microarrays from healthy donors with a history of alcohol consumption (drinker) and patients with ASH (scale bar = 400 μm, 100 μm). **J** MIST1-positive cells in the tissue sections (*n* = 9 abstainers, *n* = 5 drinkers, and *n* = 6 ASH group). All data are presented as mean ± SEM. *p < 0.05, **p < 0.01 (two-tailed unpaired Student’s test or one-way ANOVA with Dunnett’s multiple comparison).

We further evaluated the effect of MIST1 in patients with chronic liver disease using bulk RNA-sequencing data from patients with MASH in a public dataset of the Gene Expression Omnibus. *MIST1* expression levels showed a positive correlation with the MAFLD activity score (Fig. 6A). Among the score components, no significant differences in *MIST1* levels were observed according to grades of steatosis and lobular inflammation (Fig. S7A, B). However, the group with grade-2 hepatocyte swelling exhibited higher *MIST1* levels than those of the group with grades 0–1 swelling (Fig. 6B). *MIST1* expression gradually increased with increasing fibrosis scores (Fig. 6C). Immunohistochemistry of tissue microarrays showed MIST1-positive cells around the fatty area in tissues from the MASH and alcoholic steatohepatitis (ASH) groups (Fig. 6D–G, I, and J). Since ethanol consumption induces ER stress [28, 29], we also measured MIST1 expression in healthy donors who consumed alcohol, demonstrating a significantly increased MIST1-positive cell rate in cores from the livers of these donors compared to that of healthy livers from abstainers (Fig. 6E, H, and J).

## Discussion

Our data showed that sustained damage to the liver results in substantial changes in the expression of genes involved in the canonical UPR pathway. Among these genes, *MIST1* displayed a sustained increase during chronic ER stress exposure in mice and human hepatocytes. Moreover, we identified MIST1 as a putative regulator of the transcription factor TRIB3 independent of ATF4. Analysis of human samples further demonstrated that MIST1 can serve as an indicator of damage in MAFLD. Overall, our findings identify MIST1 as an ER stress-inducible gene that regulates ER stress-induced apoptosis in hepatocytes. To measure the differences in gene expression more accurately, we analyzed time-series transcriptome data from the same liver lobes of identical mice to reduce experimental variability. In previous studies in mouse models of liver injury, the damage and fibrous scarring from biopsies did not extend beyond the incision site [30], and XBP levels were reported to return to basal levels in mouse livers after partial hepatectomy within 48 h of surgery [31]. These results support that the effect of biopsy itself on ER stress levels is negligible.

A previous study showed that biweekly injections of CCl_4_ for 10 weeks resulted in a decline in macrophage accumulation, fibrogenesis, and acute damage response after six weeks, indicating a tolerance phase against continuous damage in the liver [32]. Consistently, in the present study, *Mist1* expression was sharply upregulated six weeks after CCl_4_ injury in mice. Lo et al. [20] found that ectopic induction of MIST1 in hepatocytes upregulated gene expression related to the ER–Golgi structure and ER trafficking, suggesting that MIST1 plays a protective role in injured hepatocytes. Indeed, we found that *Mist1*^KO^ mice were more susceptible to ER stress and showed higher apoptotic gene expression than WT mice, whereas no defects in the liver and cellular architecture were observed under homeostatic conditions. Of note, *Mist1*^KO^ acinar cells were also reported to be sensitive to cerulein- or alcohol-induced damage in the pancreas [33, 34]. Similar findings in the pancreas were observed in mice expressing the dominant-negative form of *Mist1* [21]. Although we did not experimentally evaluate the influence of overexpression of dysfunctional/truncated MIST1, we reason that, similar to *Mist1*^KO^ mice, such mutant mice would be susceptible to liver disease.

MIST1 expression was elevated in various damage models; however, MIST1 expression was rarely observed in the short-term stress. In the acute injury model, MIST1^+^ hepatocytes diminished 24 h after CCl_4_ injection. This reduction in MIST1 expression may be attributed to the xenobiotic metabolism and regeneration capabilities of hepatocytes, which can promote the UPR pathway. Previous studies have demonstrated that the expression of the spliced form of XBP1s, acting upstream of MIST1, is downregulated within 2 days after injury [9, 35]. However, in chronic injury models, MIST1^+^ cells remained in the pericentral region and around the central–central fibrotic septa. In addition, apoptotic cells were concentrated in the centrilobular region, suggesting that xenobiotic metabolism was not involved. Although chronic CCl_4_ administration causes hepatocyte death and inflammation in the pericentral region, MIST1^+^ pericentral hepatocytes rarely exhibited apoptotic features.

Public data showed a positive correlation between the MAFLD score and *MIST1* expression. Significantly higher *MIST1* expression were observed in patients with high balloon grades. However, there were no differences in steatosis grades. This implies that MIST1 expression is linked to the progression of MAFLD/MASH and is likely to have a greater impact on hepatic cell death than steatosis. Overall, these findings suggest that changes in MIST1 expression levels could serve as a marker of damage accumulation. Similar patterns were observed in early MAFLD/MASH and alcohol-induced fibrosis and steatosis, which often begin in the centrilobular zone [36, 37]. The CDAHFD model also exhibited a significant increase in MIST1^+^ hepatocytes, which were distributed in both the central and portal regions. Indeed, the CDAHFD-induced fibrotic septum is initiated in the periportal region and extends into the pericentral region, forming central-portal fibrosis [38].

We also found a significantly higher proportion of XBP1 and MIST1-positive cells in the livers of healthy individuals who consumed alcohol than in those who abstained. Binge ethanol consumption is known to induce ER stress and the UPR in hepatocytes, mediated by the metabolic intermediate acetaldehyde [39]. Chronic alcohol consumption can also lead to ER stress induced by reactive oxygen species [40]. In our dataset, patients with ASH tended to have a higher average proportion of MIST1^+^ hepatocytes compared to that of abstainers, although statistical significance was not reached.

TRIB3 levels were significantly different in the presence and absence of MIST1, suggesting that TRIB3 is likely a downstream molecule of MIST1. TRIB3 was reported to inhibit the production of cleaved caspase-3, the active form, by promoting the nuclear localization of pro-caspase-3 under ER stress conditions [41]. TRIB3 has been reported to be regulated by ATF4 [26, 42] and to inhibit apoptosis by inhibiting insulin-induced protein kinase B (AKT) phosphorylation [43, 44]. Under ER stress conditions, protein kinase R-like ER kinase (PERK), which is one of the UPR sensors, activates ATF4, which subsequently binds to the promoter of downstream genes such as *TRIB3* [43–46]. This PERK-dependent pathway differs from the IRE-1-dependent pathway, which regulates MIST1 activity. In the present study, the downregulation of MIST1 reduced TRIB3 expression under ER stress, while slightly increasing ATF4 expression. This suggests that the MIST1 axis rather than the ATF4 axis is dominantly involved in the regulation of TRIB3 in hepatocytes. Therefore, further investigation is needed to determine the precise mechanisms involving MIST1, ATF4, and the *TRIB3* promoter, and how these factors collaborate in ER stress-induced hepatic apoptosis. In this regard, we propose two possible hypotheses. First, MIST1 and ATF4 may form a complex to regulate TRIB3 expression. Second, MIST1 may independently activate TRIB3 because of a stronger binding affinity to the *TRIB3* promoter than ATF4 in hepatocytes.

The present study had some limitations. We observed increased expression levels of both pro- and anti-apoptotic molecules (e.g., Bmp2, Bcl2l1, Bcl2l11, and Bax) in CCl_4_-treated *Mist1*^KO^ mice (Fig. 4C); however, we did not identify the key factor explaining this pattern. While TRIB3 is known to bind directly to AKT and inhibit its phosphorylation, suggesting its involvement in the regulation of apoptosis, the specific pathways that control apoptosis remain unclear.

We expect that our results can provide data on changes in genetic expression during chronic liver injury from identical hosts. When using MIST1 as a marker for MASH, information on the patient’s use of other ER stress-inducing factors, such as alcohol or drugs, is required. In such cases, MIST1 can be used as an additional biomarker to enhance the diagnostic precision for patients with MASH and ALD. This study showed that MIST1 is a novel regulator of TRIB3 and suggested the potential of MIST1 as a marker of liver disease progression.

## Materials and methods

### Mice

WT C57BL/6 mice maintained under specific pathogen-free (SPF) conditions were used for the experiments. B6N.129S6-Bhlha15tm1Skz/J (*Mist1*^KO^) mice maintained under SPF conditions were genotyped by a standard PCR method according to the JAX® Mice genotyping protocol (Protocol 21308). The mice were anesthetized (isoflurane: 3% induction and 1% maintenance) for liver biopsy (5 g samples). CO_2_ exposure was used for euthanasia. Blood was collected via cardiac puncture immediately after euthanasia. The randomization and blinding were not used in the present study.

### Liver injury models

For CCl_4_-induced chronic liver injury, 12-week-old male WT C57BL/6 mice were administered 10% (*v*/*v*) CCl_4_ (Sigma, Burlington, MA, USA, #289116) dissolved in corn oil at 5 mg/kg body weight twice a week; control mice received the same amount of corn oil only. Liver tissues and serum were collected within 48 h of the last CCl_4_ injection at 8 weeks. Part of the medial lobe tissues were stored in RNA Later at −80 °C until bulk RNA sequencing. A portion of the remaining tissue was frozen in a deep freezer and the fatty liver content was measured using a triglyceride quantification kit (Abcam, Cambridge, UK, #ab65336) and a cholesterol HDL/LDL assay kit (Abcam, Cambridge, UK, #ab65390). *Mist1*^KO^ mice and their littermates were intraperitoneally injected with CCl_4_ under identical conditions (5 mg/kg body weight) for 8 weeks, and the control group was also intraperitoneally injected with corn oil alone. For the CDAHFD model, WT male C57BL/6 mice, 11–13-weeks-old, were fed normal chow or CDAHFD (Research Diets Inc., New Brunswick, NJ, #A06071302) for 4 weeks. For liver injury and fibrosis by obstructive cholestasis, WT C57BL/6 male mice, 8–12-weeks-old, were anesthetized (isoflurane: 3% induction and 1% maintenance) and body temperature was maintained at 37 °C with a heating pad. After shaving the abdominal fur, the skin was sterilized with an antiseptic solution and the abdominal cavity was opened with sterilized instruments. The common bile duct was tied in two knots with non-absorbable suture (Ailee, Busan, South Korea, sk434), and the peritoneum was sutured with absorbable suture (Ethicon, Cincinnati, OH, USA W9113). The skin was sutured with a non-absorbable suture and the povidone-iodine solution was applied around the suture. After surgery, the surgical wound. For tunicamycin-induced liver injury, tunicamycin was dissolved in DMSO to make a 40 mg/ml stock solution. WT C57BL/6 mice were administered 1% (*v*/*v*) stock solution dissolved in 1× phosphate-buffered saline (PBS) at a dose of 2 mg/kg body weight once, and control mice were administered 1× PBS. Liver biopsy specimens were obtained at each time point.

### Immunostaining

Human tissue microarray slides from patients with metabolic dysfunction-associated steatohepatitis (MASH) were obtained from Xenotech (TMA.MASH, Xenotech, Kansas City, KS, USA, Lot No. 2210214) for immunohistochemistry. Samples were deparaffinized and sequentially rehydrated using a descending graded series (100%, 95%, and 70%) of ethanol. Antigen retrieval (Agilent, Santa Clara, CA, USA, #S1699) was performed using a pressure cooker. After cooling on ice for at least 1 h, the sections were incubated in 3% H_2_O_2_ for 30 min to block endogenous peroxidase. Sections were washed twice with PBS and incubated with a serum-free protein block (Agilent, Santa Clara, CA, USA, #X0909) for 1–2 h at room temperature to reduce non-specific signals. Treatment with M.O.M (Vector Laboratories, Newark, CA, USA, #BMK-2202) reagent for 1 h was performed using mouse primary antibodies. The sections were then incubated with primary antibodies at 4 °C overnight (Table S1). After three washes in PBS, the sections were incubated with a horseradish peroxidase-conjugated secondary antibody (Agilent, Santa Clara, CA, USA, #K4001) for 15 min at room temperature. DAB (Agilent, Santa Clara, CA, USA, #K3468) was used for antibody development and Mayer’s hematoxylin (Agilent, Santa Clara, CA, USA, #3309) was used for counterstaining. Each experiment was performed at the same time as DAB development. For immunofluorescence, the primary antibodies (Table S1) were detected using fluorophore-conjugated (Alexa488, Cy3, or Cy5) secondary antibodies. Immunohistochemical slides were scanned using Easy Scan (Motic, Xiamen, China). DAB-positive nuclei in the tissue sections were measured using the digital image analysis program Qupath (University of Edinburgh, Edinburgh, Scotland). The liver tissue sections of mice were evaluated for the degree of inflammation via the detection of macrophage recruitment, including Kupffer cells related to disease progression [47], using F4/80 immunohistochemistry staining.

### RNA-sequencing data and bioinformatic analysis

We preprocessed the raw reads obtained from the sequencer to remove low-quality and adapter sequences before analysis, and aligned the processed reads to the *Mus musculus* (mm10) reference genome using HISAT v2.1.0. HISAT utilizes two types of indices for alignment, including a global whole-genome index and tens of thousands of small local indices. The reference genome and annotation data were downloaded from the UCSC Table Browser (http://genome.uscs.edu). Transcript assembly and relative abundance estimation (according to read counts) were performed using StringTie v.1.3.4d. Differentially expressed genes (DEGs) between CCl_4_-treated and control mice were identified using the estimates of abundance for each gene in the samples according to criteria of |fold change | ≥ 2 and raw *p* < 0.05. Genes with one or more read count values were excluded. The filtered data were log2-transformed and subjected to relative log expression normalization. For the DEG set, hierarchical clustering analysis was performed using complete linkage and Euclidean distance as similarity measures. Gene enrichment, functional annotation, and pathway analyses for significant genes were performed using gProfiler (https://biit.cs.ut.ee/gprofiler/orth) and the Kyoto Encyclopedia of Genes and Genomes pathway database (http://www.genome.jp/kegg/pathway.html). We explored the binding of MIST1 to candidate target gene sequences in HepG2 cells that had been modified with CRISPR. The data were downloaded from the Encyclopedia of DNA Elements website (https://www.encodeproject.org) (ENCSR888QFJ) and analyzed using ChiPseeker ver.4.2.1 with the annotation database TxDb.Hsapiens. UCSC.hg38.known Gene. Only binding sites residing within ±3 kb of the transcriptional start site of each target gene were considered in the analysis. Integrative Genomics Viewer (version 2.16.0) was used to visualize the alignment with the ChIP-seq peak and annotation sequence [48].

### Statistical analysis

To evaluate significance, at least three animals were used in each group. All statistical analyses were conducted using GraphPad Prism 9 software (https://www.graphpad.com/). Data are presented as the mean ± SEM. Statistical significance was determined using unpaired Student’s *t*-test or one-way ANOVA with Dunnett’s multiple comparison test; *p* < 0.05 was considered significant.

## Supplementary information

Supplemental material

Uncropped Western blot

## Data Availability

The sequencing data used in this study have been deposited in the database of the Korean Nucleotide Archive (KONA) at https://kobic.re.kr/kona/. BioProject Accession ID: KAP230676.
