# Supplementary material for: MIST1 regulates endoplasmic reticulum stress-induced hepatic apoptosis as a candidate marker of fatty liver disease progression
Source: Cell Death Dis. 2024 Nov 8;15(11):805. doi: 10.1038/s41419-024-07217-0 (PMC11549289; doi:10.1038/s41419-024-07217-0)
Supplement: Supplementary file 1 — Supplemental material [file 41419_2024_7217_MOESM1_ESM.docx]

**Supplementary data**

**MIST1 regulates endoplasmic reticulum stress-induced hepatic apoptosis as a candidate marker of fatty liver disease progression**

Sumin Hur, Haengdueng Jeong, Keunyoung Kim, Kwang H. Kim, Sung Hee Kim, Yura Lee, Ki Taek Nam

**Supplementary material**

**Cell line and culture**

The human liver cancer cell line HepG2 was maintained in Dulbecco’s modified Eagle medium (Cytiva, Marlborough, MA, USA, #SH30243.01) containing 10% heat-inactivated fetal bovine serum (FBS) and 1% streptomycin/penicillin at 37℃ in a humidified 5% CO_2_ incubator. Cells were incubated in 0.5 μg/ml tunicamycin in the culture medium containing 10% FBS for 3 h to 48 h to induce ER stress.

**Immunoblotting**

Proteins were extracted from liver specimens and cell lines using protein lysis buffer (20 mM HEPES [pH 7.0], 0.15 M NaCl, 10% glycerol, 1% Triton X-100, 1 mM EDTA, 1 mM EGTA, 10 mM β-phosphoglycerate) containing protease and phosphatase inhibitor cocktails (Thermo, Waltham, MA, USA). All lysates were collected by centrifugation (13,000 rpm, 15 min) and boiled in 1× SDS-PAGE sample buffer after measurement of protein concentrations. Subsequently, 20 μg of the protein sample was separated by SDS-PAGE and transferred to a polyvinylidene fluoride membrane (Millipore, Billerica, MA, USA). The membrane was then incubated with primary antibodies overnight at 4°C. After washing, the membranes were incubated with secondary antibodies for 1 h at room temperature. After washing, a chemiluminescent substrate (Thermo, Waltham, MA, A38554) was used to detect the signal. The list of antibodies is provided in Table S1.

**Histological analysis**

After perfusion with cold 1× PBS, parts of the medial lobe of the liver were collected and immersed in 4% paraformaldehyde (PFA) at room temperature overnight. After fixation, the liver tissues were embedded in paraffin. Paraffin-embedded blocks were sectioned at 5 μm width and then the sections were stained with hematoxylin & eosin, Picro-Sirius Red (ScyteK, Logan, UT, USA), collagen staining (3HELIX, Salt Lake City, UT, USA, #R-CHP), and terminal deoxynucleotidyl transferase dUTP nick end labeling (TUNEL) staining (Elabscience, Houston, TX, USA, #E-CK-A321). Snap-frozen left lateral lobe tissue blocks were cut in 10-μm-wide sections for Oil red O staining (Oil Red O Stain Kit, ScyteK, Logan, UT, USA). The measurements were performed according to the manufacturer protocols.

**Serum protein level**

Blood samples were collected and incubated at room temperature for 30 min. After incubation, the serum was separated from the blood cells by centrifugation at 1000 ×*g* and 4°C. The levels of aspartate aminotransferase (AST), alanine aminotransferase (ALT), and albumin were determined using dry-chem slides (478806, 413907, and 158304, respectively; FUJI film).

***MIST1* knockdown *in vitro***

HepG2 cells were seeded in 6-well plates at 1 × 10^5^ cells/well and incubated for 2 days before transfection with human *MIST1* siRNA (FlexiTube GeneSolution 4 siRNAs, Qiagen, Hilden, Germany, GS168620) or negative control siRNA (AllStar Negative Control siRNA; Qiagen, Hilden, Germany, #1027280) using HiPerFect Transfection Reagent (Qiagen, Hilden, Germany, #301705) according to the manufacturer’s protocol. One day after transfection, the growth medium was changed to Opti-MEM (#31985062; Gibco, Waltham, MA, USA) without FBS for starvation.

**RNA extraction and quantitative reverse transcription real-time PCR**

For RNA extraction, liver samples dipped in RNAlater (Thermo, Waltham, MA, USA, #AM7020) were homogenized and transferred to 1.5 ml conical tubes containing TRIzol solution (Invitrogen, #15596018) in accordance with the manufacturer’s protocol. cDNAs were synthesized from 1 μg samples after treatment with DNase (Takara, Kusatsu, Japan, #2270A) using the ImProm-II™ reverse transcription system (Promega, Madison, WI, USA, #A3800). Quantitative PCR was conducted using POWER SYBR Green Master Mix (Takara, Kusatsu, Japan) under the following reaction protocol: 95℃ for 3 min, followed by 40 cycles of 95℃ for 10 s and 60℃ for 30 s for melting curve analysis. The primers are listed in Table S2.

**Immunocytochemistry**

For immunocytochemistry, HepG2 cells (2 × 10^4^ cells/well) were planted on poly-l-lysine (Sigma-Aldrich, St. Louis, MO, USA)-coated 24-well chamber slides and fixed in 4% PFA for 15 min. The fixed cells were washed three times with ice-cold PBS and incubated with 0.25% Triton X-100 in PBS for 15 min for permeabilization. Cells were incubated with 1% bovine serum albumin for 30 min to block non-specific binding, followed by primary antibody treatment at 4°C overnight (Table S1). After two washes in ice-cold PBS, the primary antibodies were detected using fluorophore-conjugated anti-IgG (Thermo, Waltham, MA, USA), followed by DAPI staining.

**Luciferase assay**

A putative MIST1-binding region (Chr20 380254-381174) was restricted from the commercial TRIB3 promoter vector (Active Motif, Carlsbad, CA, USA, S708065). The restricted insert was then inserted into a promoterless firefly luciferase basic vector (Promega, Madison, WI, USA, E6651) using *Xho*I/*Bgl*II. The HepG2 cell line was seeded in a 6-well plate at 1 × 10^6^ cells per well at passage 3. Once the cell confluency reached ~70%, the medium was replaced with Opti-MEM(#31985062; Gibco, Waltham, MA, USA), and the cells were co-transfected overnight at 37°C using Lipofectamine™ 3000 Transfection Reagent (Thermo Fisher Scientific, Waltham, MA, USA, L3000001) with MIST1 siRNA (75 pmol) (FlexiTube GeneSolution 4 siRNAs, Qiagen, Hilden, Germany, GS168620) and TRIB3-luciferase vector and pRL Renilla control (Promega, Madison, WI, USA, E2261) (3 µg and 1.5 µg each). The next day, the medium was replaced with growth medium containing 0.5 μg/ml tunicamycin. After 24 and 48 h, the cells were washed with PBS and lysed with passive lysis buffer (Promega, Madison, WI, USA, E194A). Luciferase activity was measured using the dual-Luciferase assay kit (Promega, Madison, WI, USA, E1910) with the Centro XS3 LB960 instrument (EG & G Berthold, Centro XS3, Germany). Relative levels of luciferase activity were normalized to the levels of Renilla luciferase activity.

**
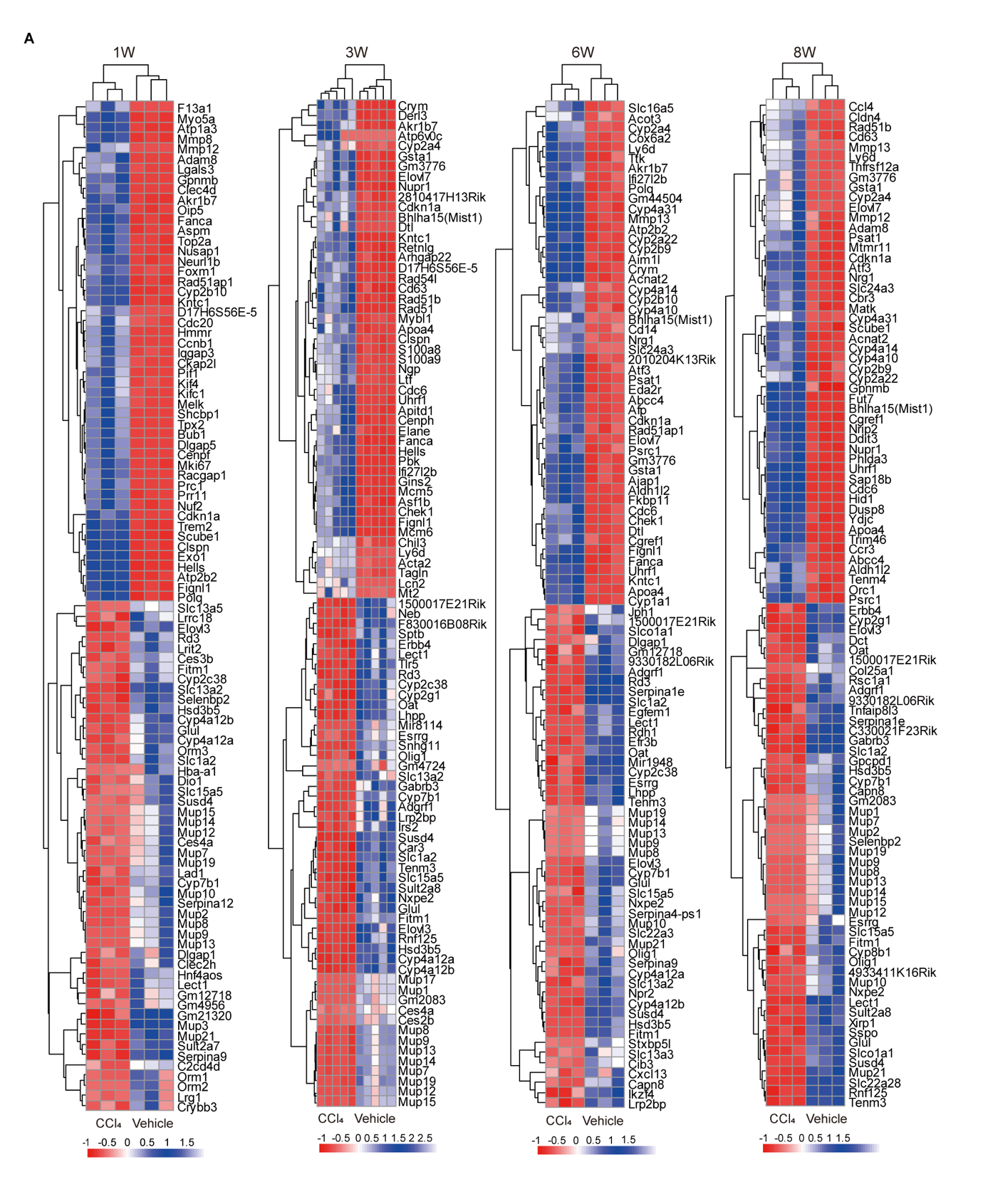
Supplementary Fig. S1.** (A) Heatmap of the top 50 up- or downregulated genes in mice treated with CCl_4_ at 1, 3, 6 and 8 weeks.

**
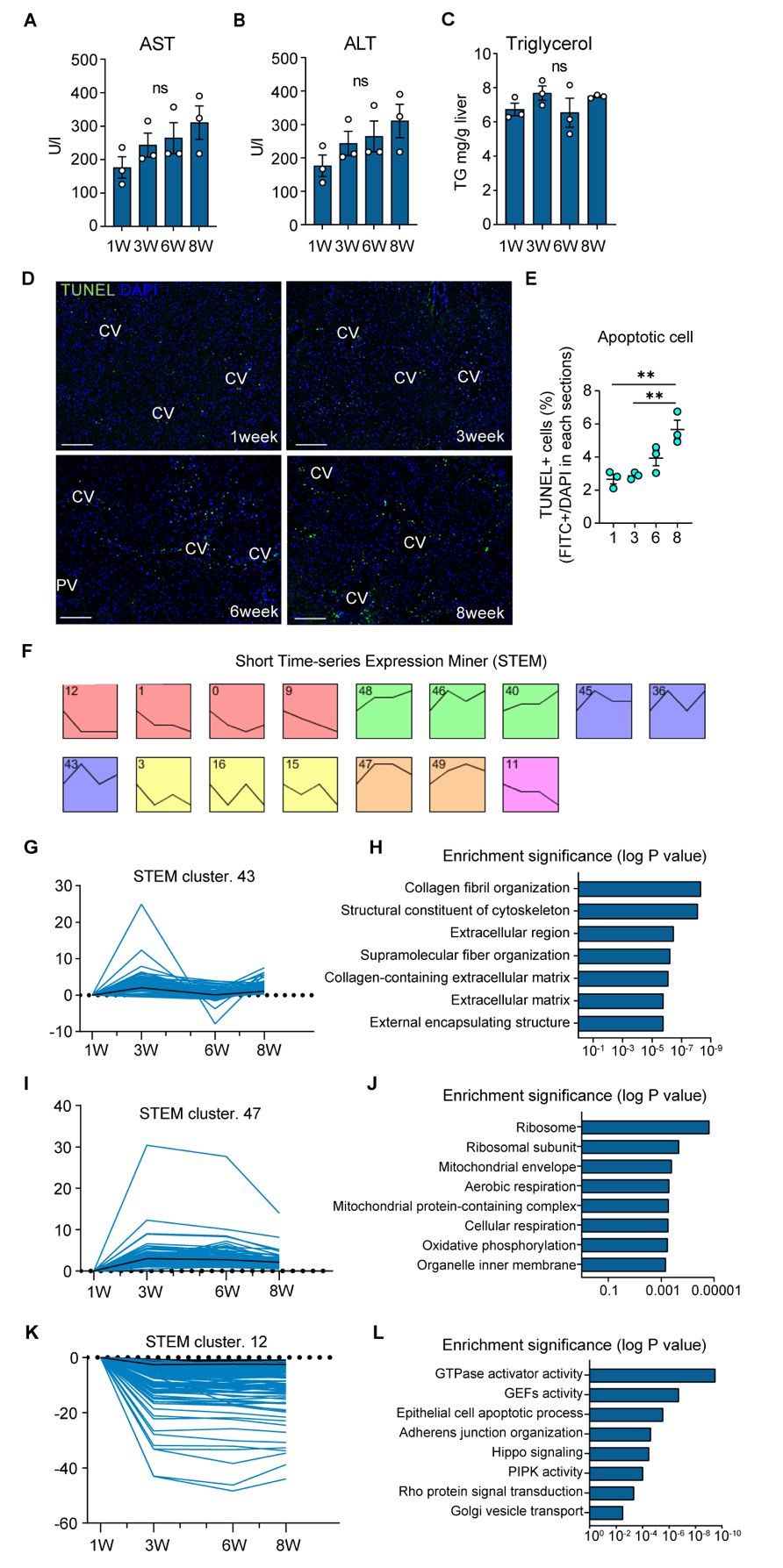
**

**Supplementary Fig. S2.** (A, B) Levels of blood serum transaminases; n = 3 per group. (C) Triglyceride content in the caudate lobe; n = 3 per group. (D) Representative images of TUNEL staining at each time point (Scale bar, 400 μm). (E) Percentile of TUNEL-positive nuclei at each time point in liver tissue sections (n = 3); numbers in the x-axis indicate weeks of CCl_4_ treatment. Data are presented as mean ± SEM. ***p* < 0.01 (one-way ANOVA with Dunnett’s multiple comparison). (F) Representative patterns of the time-series graph for each short time-series expression miner (STEM) cluster. Clusters are ordered based on the number of genes and profiles are ordered by significance. Clusters of seven lineages (red, green, blue, purple, yellow, orange, and pink) are classified based on similarity. (G, I, K) Trajectory plot of gene set cluster 43, 12, and 47 identified by STEM analysis (red line = *Mist1* expression). (H, J, L) Top eight significantly enriched Gene Ontology terms for cluster 43, 12, and 47.

**
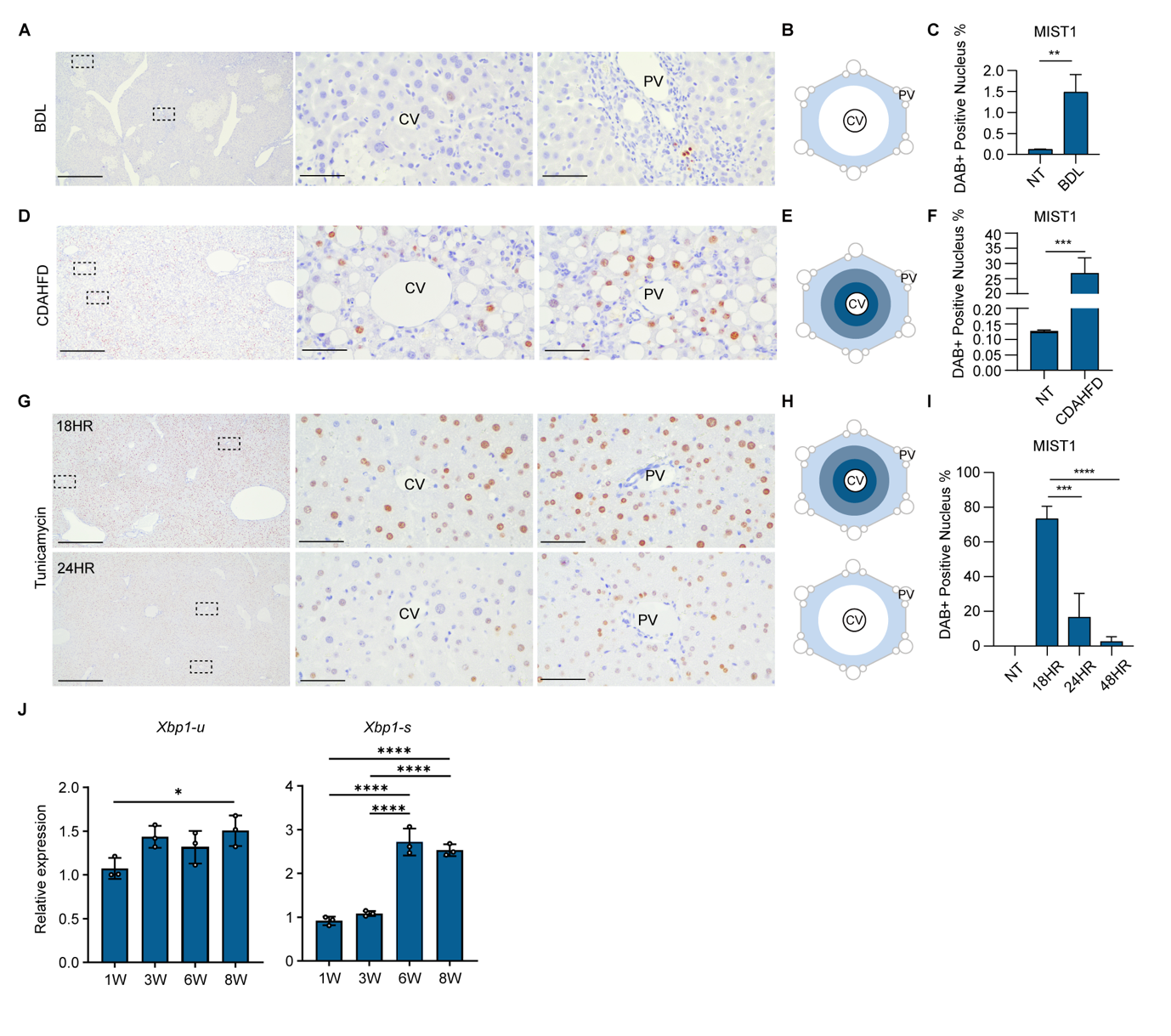
Supplementary Fig. S3.** (A) Immunohistochemistry for MIST1 in the liver of the bile duct-ligated (BDL) mouse model. Liver samples were collected 2 weeks after BDL. (CV = central vein, PV = portal vein). Scale bars: 400 μm, 100 μm. (B, E, H) Distribution of MIST1-positive cells (colored area). (C, F, I) Percentage of MIST1-positive cells for each time point in the whole-tissue sections (n = 3). (D) Immunohistochemistry for MIST1 in the liver of the choline deficient l-amino acid-deficient, high-fat diet (CDAHFD) mouse model. (G) Immunohistochemistry for MIST1 in the liver after induction of ER stress by tunicamycin administration. Liver samples were collected at 18 and 24 h after tunicamycin intraperitoneal injection (CV = central vein, PV = portal vein, NT = not treated); n = 3 per group. (J) Relative mRNA levels of *Xbp1-u* and *Xbp1-s* over time during CCl_4_ administration. *Xbp1-u* and *Xbp1-s* indicate the unspliced and spliced forms of *Xbp1*, respectively. n = 3 per group. All data are presented as mean ± SEM. **p* < 0.05, ***p* < 0.01, ****p* < 0.001, *****p* < 0.0001 (two-tailed unpaired Student’s t-test or one-way ANOVA with Dunnett’s multiple comparison).

**
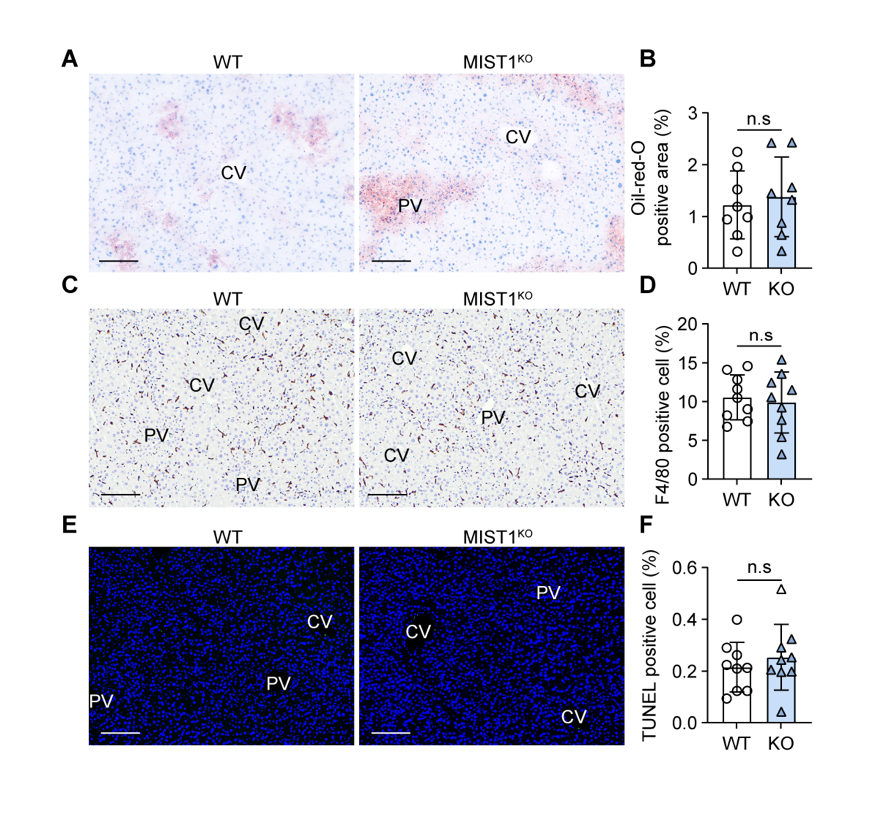
**

**Supplementary Fig. S4**. (A, C, E) Oil red O, F4/80 immunohistochemistry and TUNEL immunofluorescence in Mist1KO mice and wild-type (WT) littermates treated with corn oil as the control. (B) Lipid droplet accumulation, (D) macrophage recruitment, and (F) apoptotic cell counts in tissue sections; n = 9 per group. (CV, central vein; PV, portal vein). Scale bars: 100 μm. All data are presented as mean ± SEM (two-tailed unpaired Student’s t-test).

**
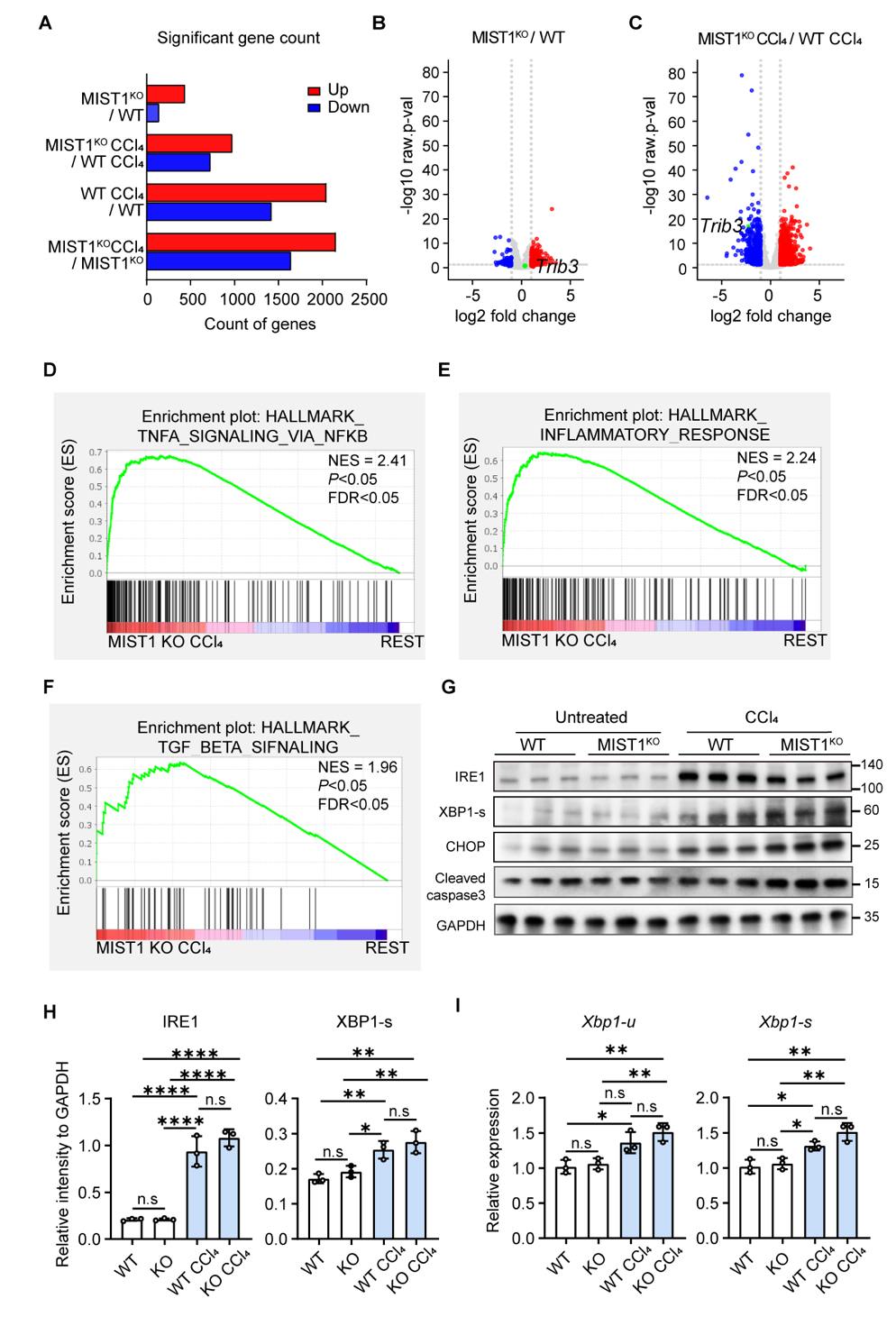
**

**Supplementary Fig. S5.** (A) Numbers of significantly up- or down-regulated genes in the *Mist1*^KO^ and wild-type mouse liver based on RNA-sequencing data. (*p* < 0.05, log2 fold change > 2). (B) Volcano plot of RNA-seq datasets from the non-treated *Mist1*^KO^ and wild-type mice. (C) Volcano plot of RNA-seq datasets from CCl_4_-treated *Mist1*^KO^ and wild-type mice. (D–F) Gene set enrichment analysis comparing CCl_4_-treated *Mist1*^KO^ mice and the other groups (non-treated *Mist1*^KO^, non-treated wild-type, and CCl_4_-treated wild-type mice) at 8 weeks. (NES = normalized expression score, p = nominal p-value, FDR = false discovery rate q-value). (G) Western blot data for ER stress molecules (IRE1, XBP-1, CHOP) and an apoptosis marker (cleaved caspase-3) in liver specimens from CCl_4_-treated control (WT) and *Mist1*^KO^ groups. (H) Relative protein level of IRE1 and XBP1-s in liver specimens from CCl_4_-treated control (WT) and *Mist1*^KO^ groups; n = 3 per group. (I) Relative mRNA levels of *Xbp1-u* and *Xbp1-s* in liver specimens from CCl_4_-treated control (WT) and *Mist1*^KO^ mice. *Xbp1-u* and *Xbp1-s* indicate unspliced *Xbp1* and spliced *Xbp1*, respectively. n = 3 per group. All data are presented as mean ± SEM. **p* < 0.05, ***p* < 0.01, *****p* < 0.0001 (one-way ANOVA with Dunnett’s multiple comparison).

**
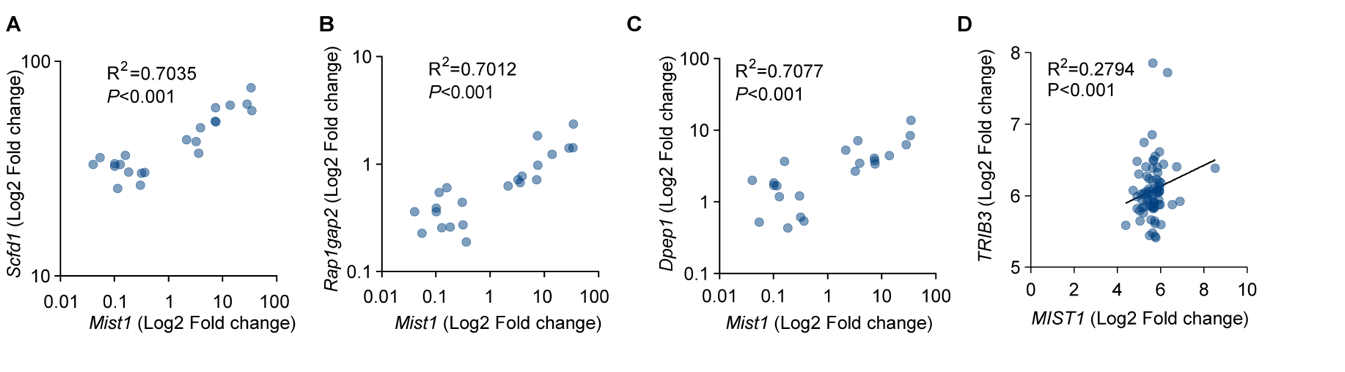
Supplementary Fig. S6.** (A-C) Scatterplots of the correlation between the normalized expression of candidate genes targeted by MIST1 in mice. (D) Scatterplots of the correlation between the normalized expression of candidate genes targeted by MIST1 in MASH patients and healthy donors.

**
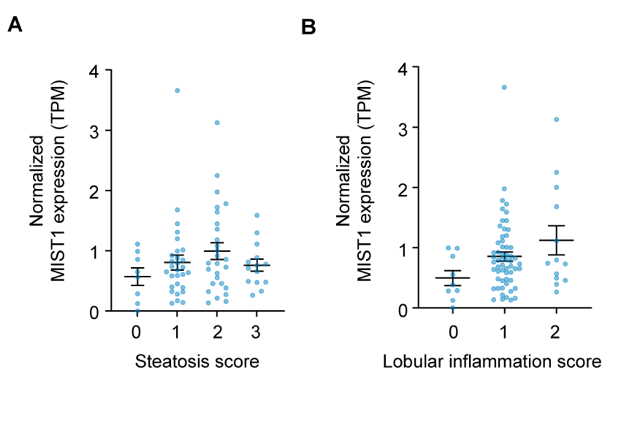
**

**Supplementary Fig. S7.** (A) Scatterplots showing the correlation between the expression of MIST1 and steatosis score in patients with MAFLD. (B) Scatterplots showing the correlation between the expression of MIST1 and lobular inflammation score in patients with MAFLD.

**Supplementary Table S1. List of antibodies.**

| Antibody | Catalog/clone number | Source |
| --- | --- | --- |
| MIST1/bHLHa15 (D7N4B) XP Rabbit mAb | 14896S | Cell Signaling Technology |
| XBP1-s | 12782S | Cell Signaling Technology |
| IRE1 | 3294S | Cell Signaling Technology |
| CHOP | 2895S | Cell Signaling Technology |
| HNF4α | Sc-374229 | Santa Cruz Biotechnology |
| KI67 | ab1667 | Abcam, Cambridge, UK |
| GAPDH | ab181602 | Abcam, Cambridge, UK |
| TRIB3 | Ab137526 | Abcam, Cambridge, UK |
| Cleaved caspase-3 | 9664 | Cell Signaling Technology |
| HRP-conjugated anti-rabbit | 7074P2 | Cell Signaling Technology |
| HRP-conjugated anti-mouse | 7076P2 | Cell Signaling Technology |

**Supplementary Table S2.** List of primers used in quantitative reverse transcription real-time PCR.

| Gene | Forward sequence (5′–3′) | Reverse sequence (5′–3′) |
| --- | --- | --- |
| *MIST1* | CGGATGCACAAGCTAAATAAC G | GCCGTCAGCGATTTGATGTAG |
| *DDIT3/CHOP (NM_004083)* | GGTATGAGGACCTGCAAGAGGT | CTTGTGACCTCTGCTGGTTCTG |
| *ATF4* | TTCTCCAGCGACAAGGCTAAGG | CTCCAACATCCAATCTGTCCCG |
| *XBP1-u* | CAGACTACGTGCACCTCTGC | CTGGGTCCAAGTTGTCCAGAAT |
| *XBP1-s* | GCTGAGTCCGCAGCAGGT | CTGGGTCCAAGTTGTCCAGAAT |
| *Xbp1-u* | CATCACTGCCACCCAGAAGACTG | ATGCCAGTGAGCTTCCCGTTCAG |
| *Xbp1-s* | GCTGAGTCCGCAGCAGGT | CAGGGTCCAACTTGTCCAGAAT |
| *GAPDH* | CCACTCCTCCACCTTTGA | ACCCTGTTGCTGTAGCCA |
| *Gapdh* | CATCACTGCCACCCAGAAGACTG | ATGCCAGTGAGCTTCCCGTTCAG |
