# Supplementary material for: MIST1 regulates endoplasmic reticulum stress-induced hepatic apoptosis as a candidate marker of fatty liver disease progression
Source: Cell Death Dis. 2024 Nov 8;15(11):805. doi: 10.1038/s41419-024-07217-0 (PMC11549289; doi:10.1038/s41419-024-07217-0)

Full and uncropped western blot for Figure 4F
Lanes 1, 2, 3, 4 are on the figure

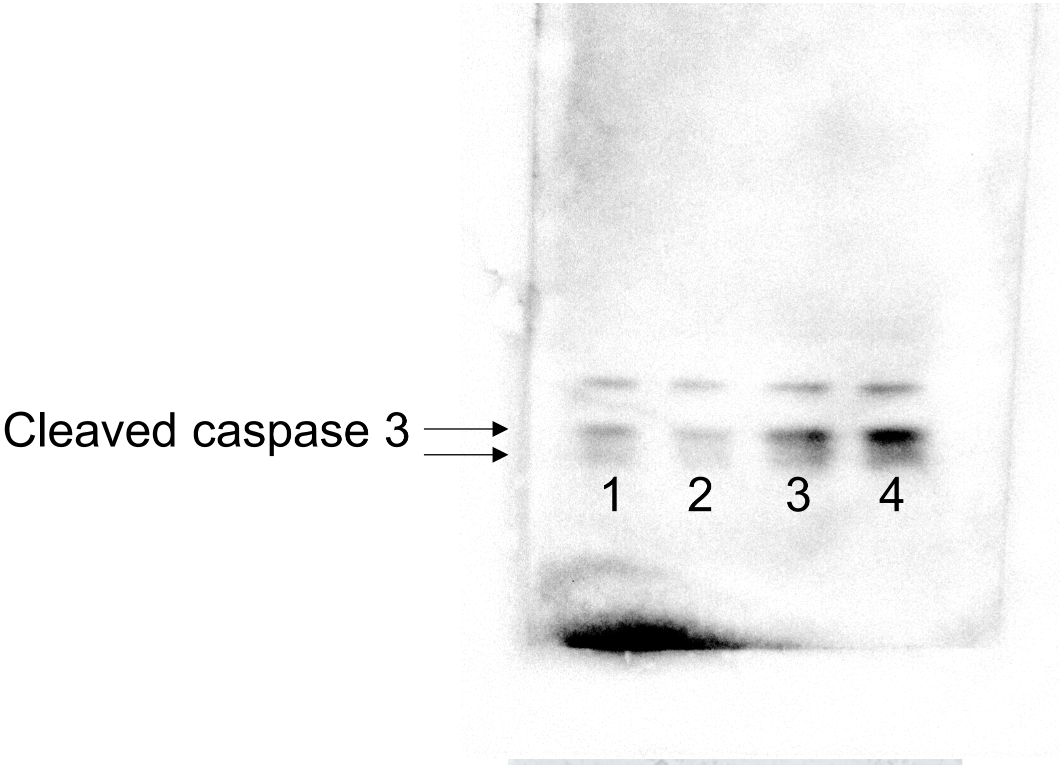

Full and uncropped western blot for Figure 4F
Lanes 1, 2, 3, 4 are on the figure

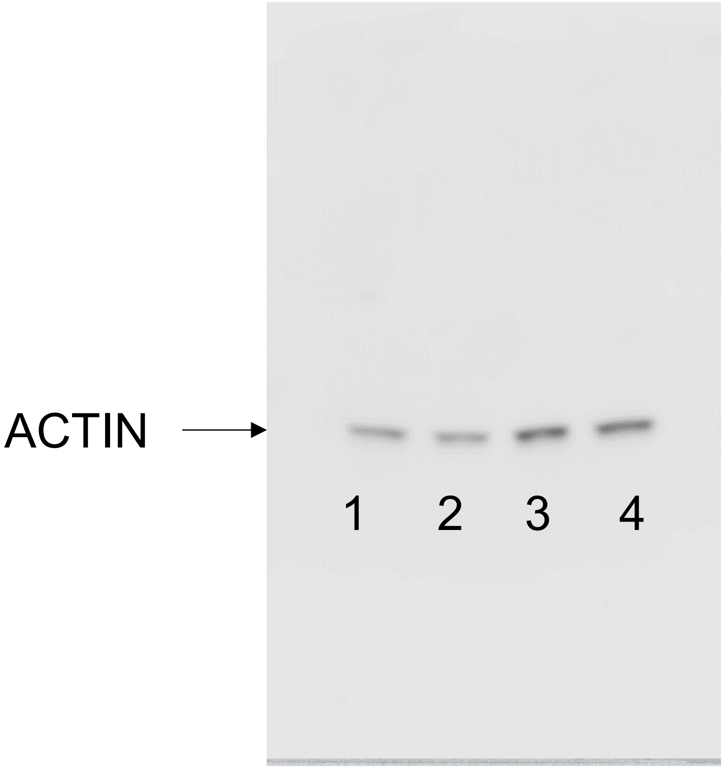

Full and uncropped western blot for Figure 5E
Lanes 1, 2, 3, 4 are on the figure

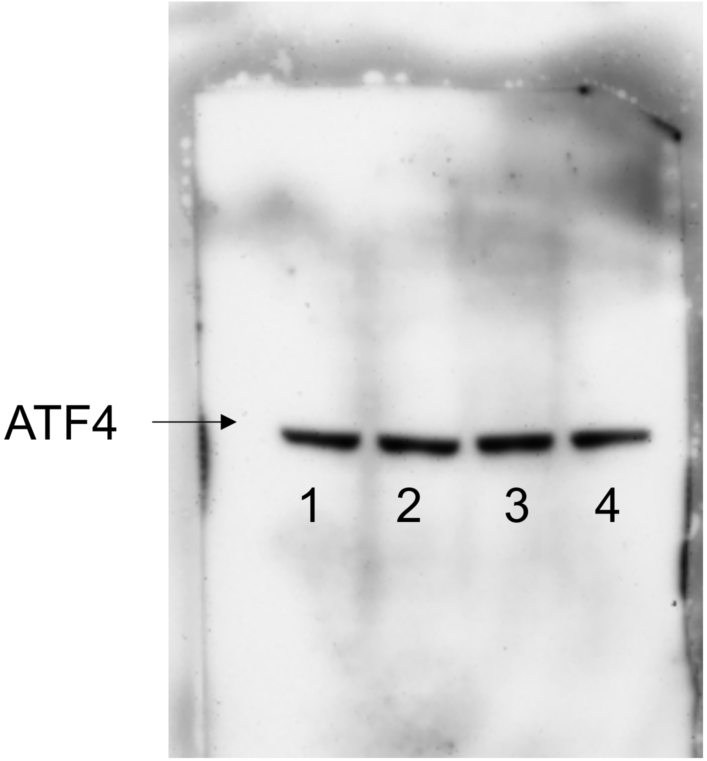

Full and uncropped western blot for Figure 5E
Lanes 1, 2, 3 4 are on the figure

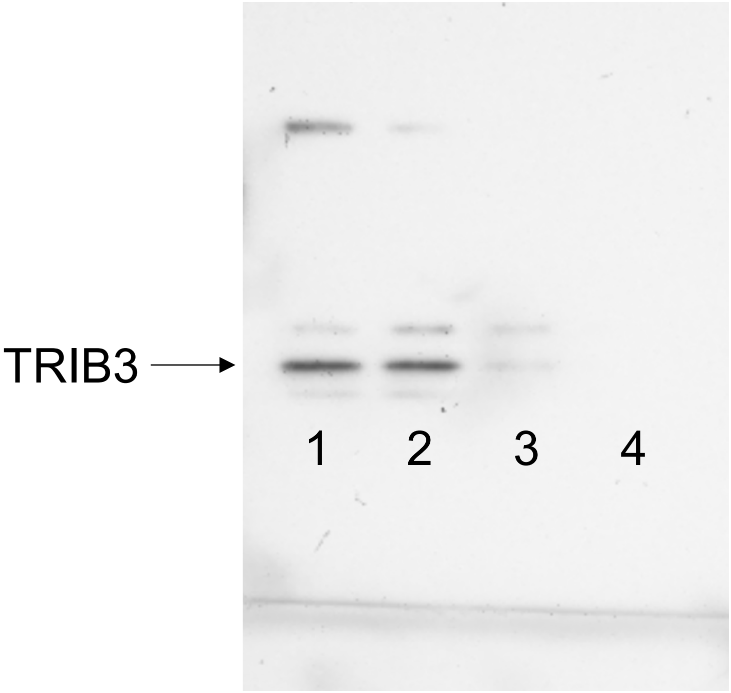

Full and uncropped western blot for Figure 5E
Lanes 1, 2, 3, 4 are on the figure

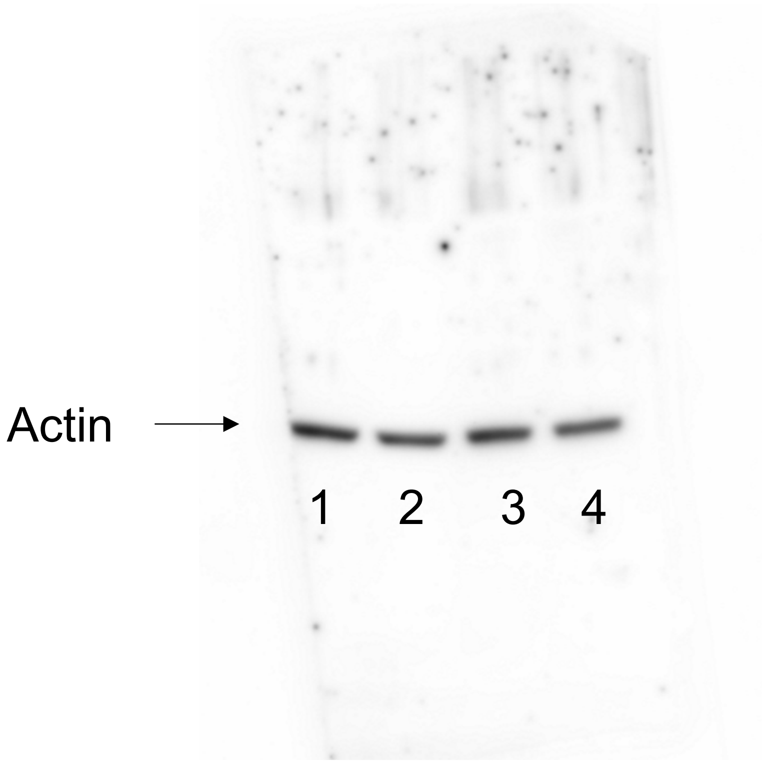

Full and uncropped western blot for Figure 5H
Lanes 1, 2, 3 are on the figure

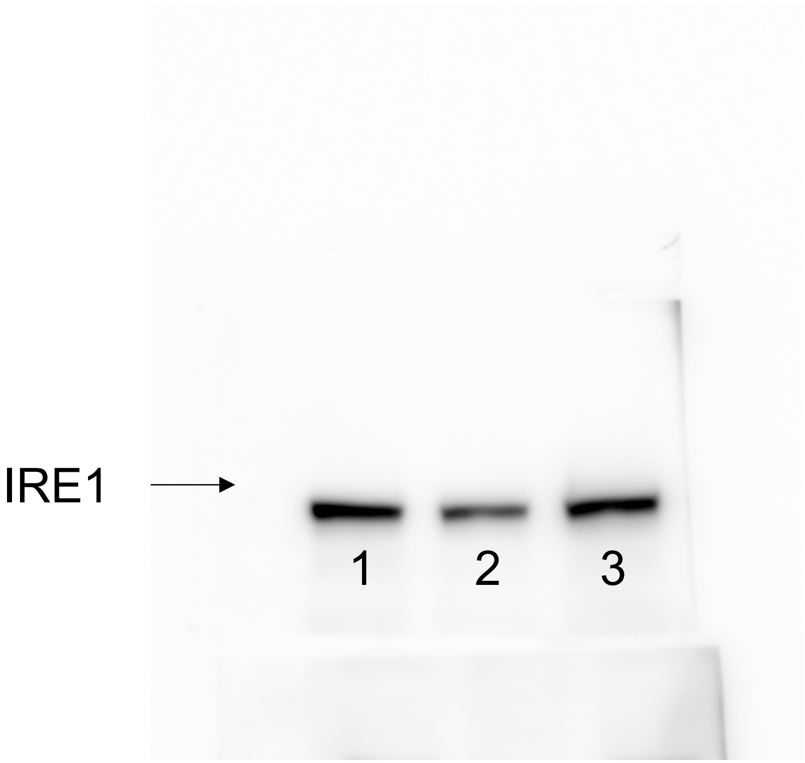

Full and uncropped western blot for Figure 5H
Lanes 1, 2, 3 are on the figure

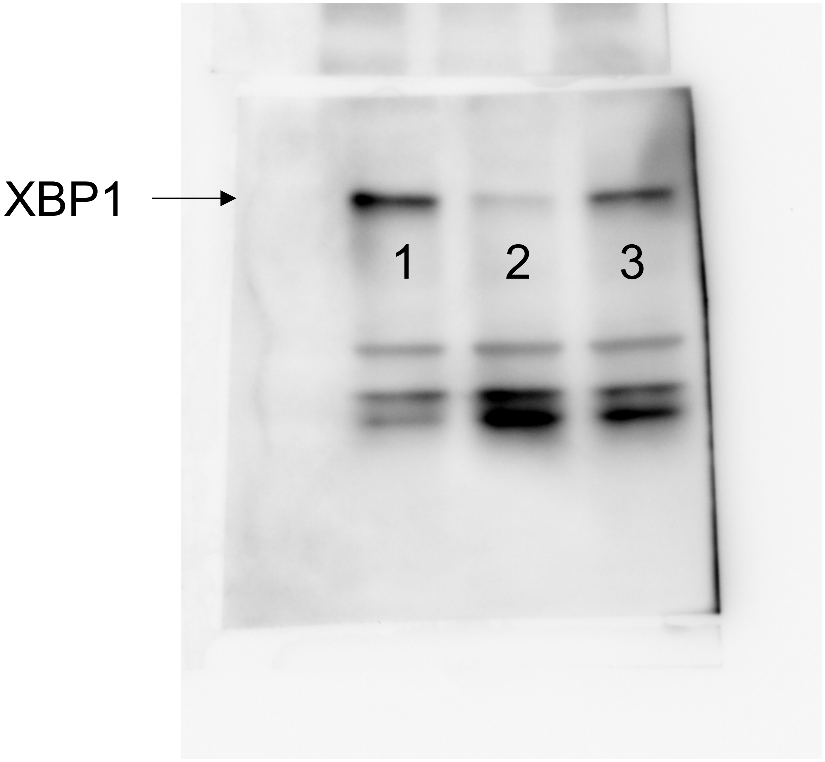

Full and uncropped western blot for Figure 5H
Lanes 1, 2, 3 are on the figure

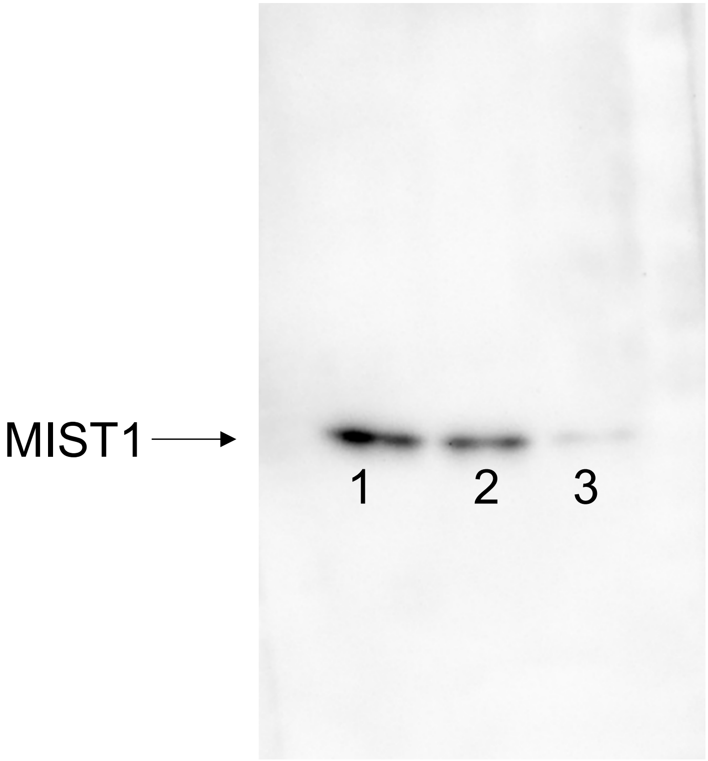

Full and uncropped western blot for Figure 5H

Lanes 1, 2, 3 are on the figure

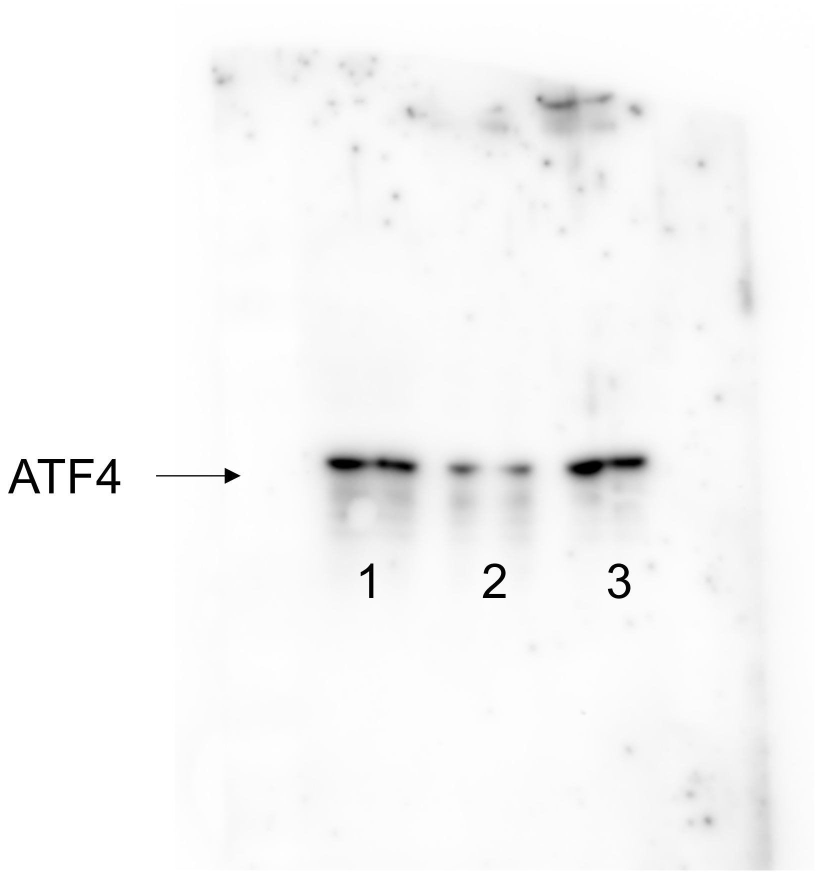

Full and uncropped western blot for Figure 5H
Lanes 1, 2, 3 are on the figure

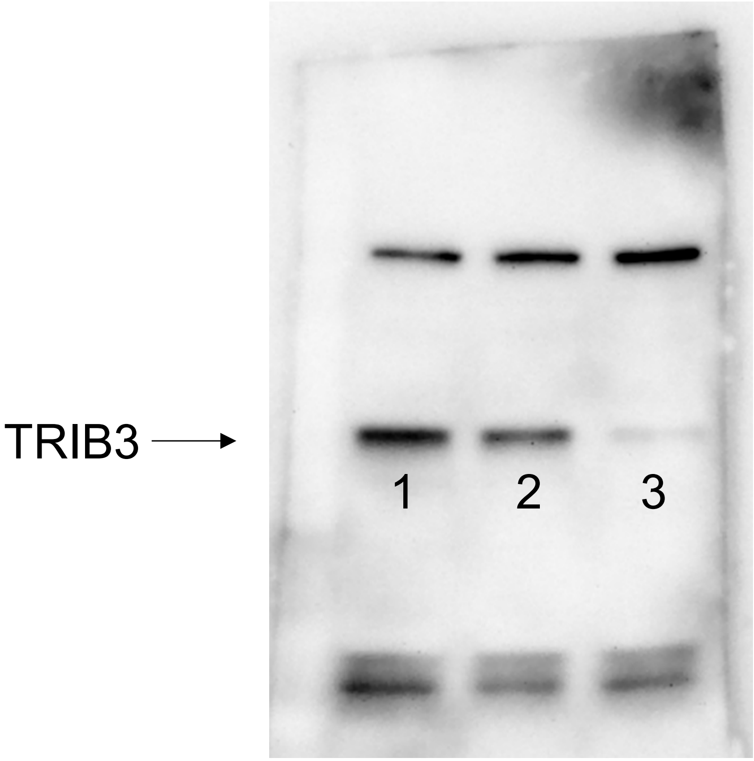

Full and uncropped western blot for 5H
Lanes 1, 2, 3 are on the figure

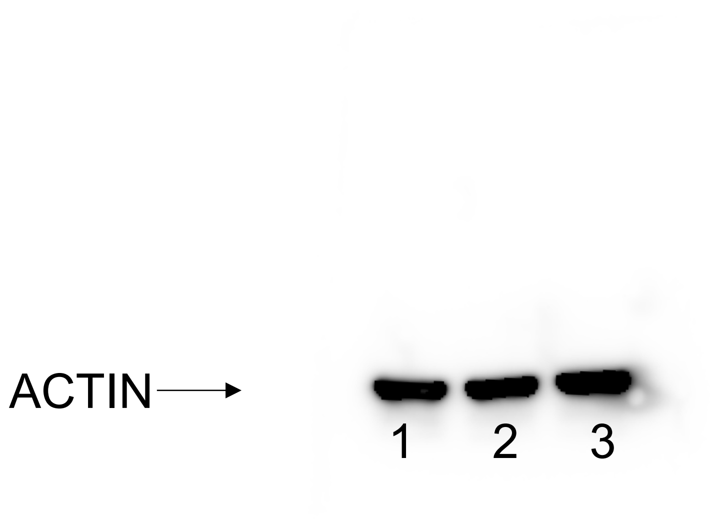

Full and uncropped western blot for Supplementary Figure 5G
Lanes 1, 2, 3, 4, 5, 6, 7, 8, 9, 10, 11, 12 are on the figure

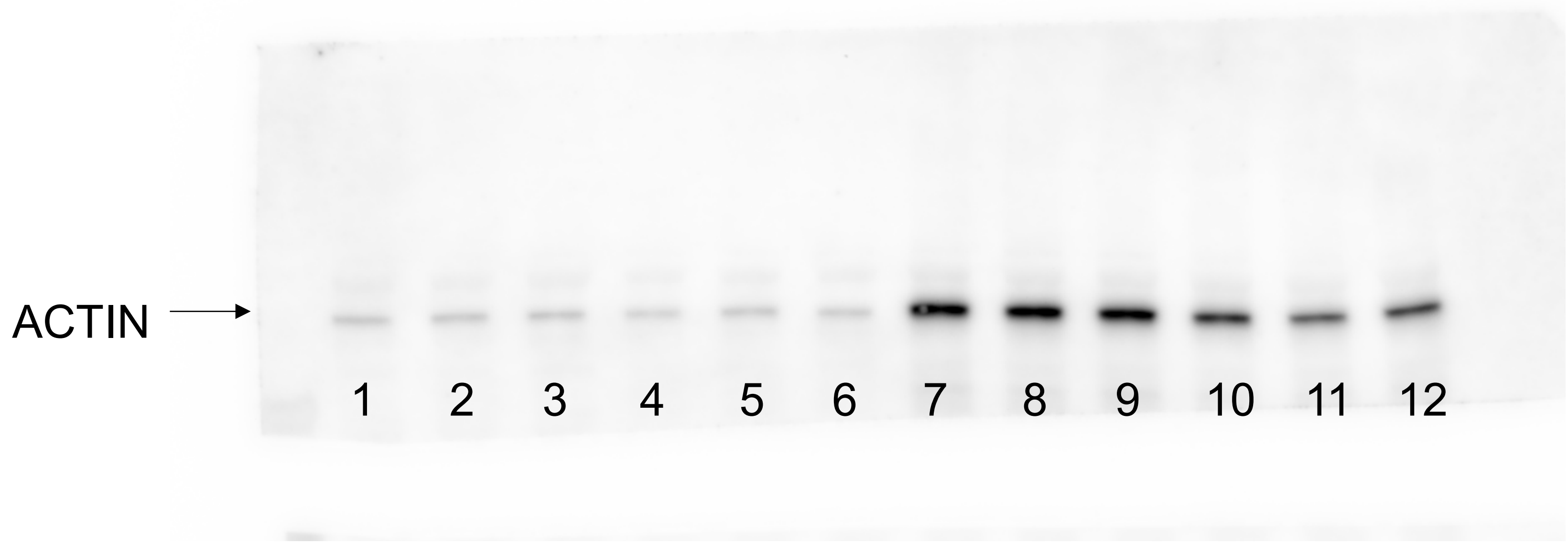

Full and uncropped western blot for Supplementary Figure 5G
Lanes 1, 2, 3, 4, 5, 6, 7, 8, 9, 10, 11, 12 are on the figure

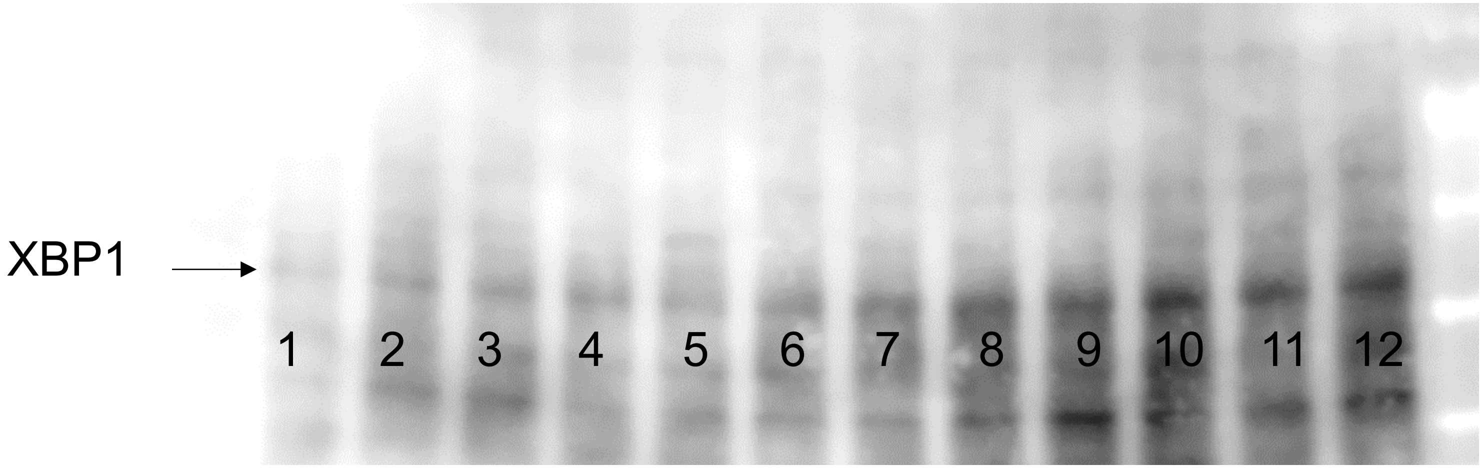

Full and uncropped western blot for Supplementary Figure 5G
Lanes 1, 2, 3, 4, 5, 6, 7, 8, 9, 10, 11, 12 are on the figure

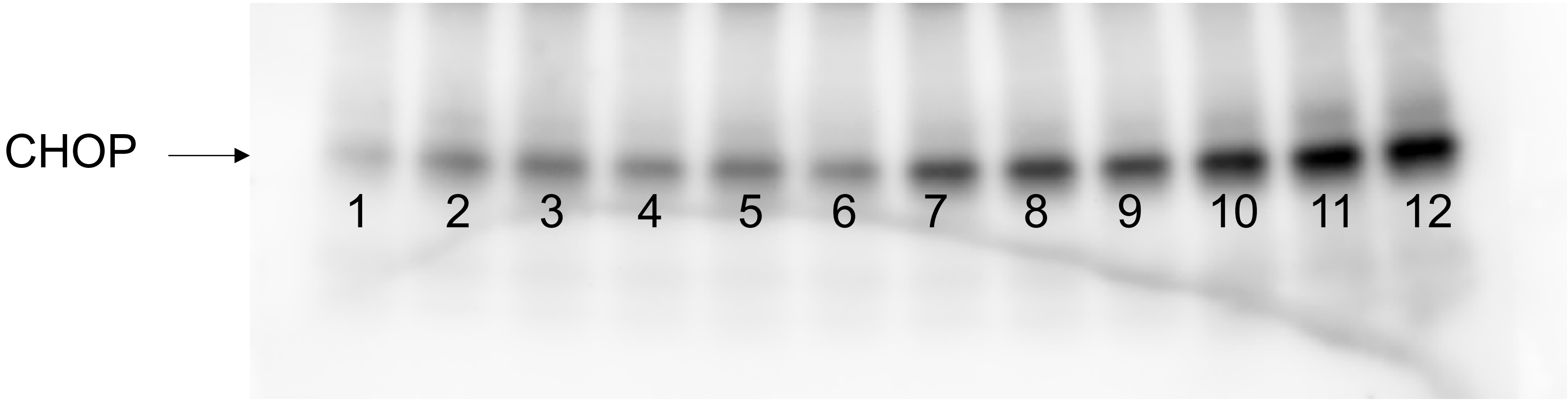

Full and uncropped western blot for Supplementary Figure 5G
Lanes 1, 2, 3, 4, 5, 6, 7, 8, 9, 10, 11, 12 are on the figure

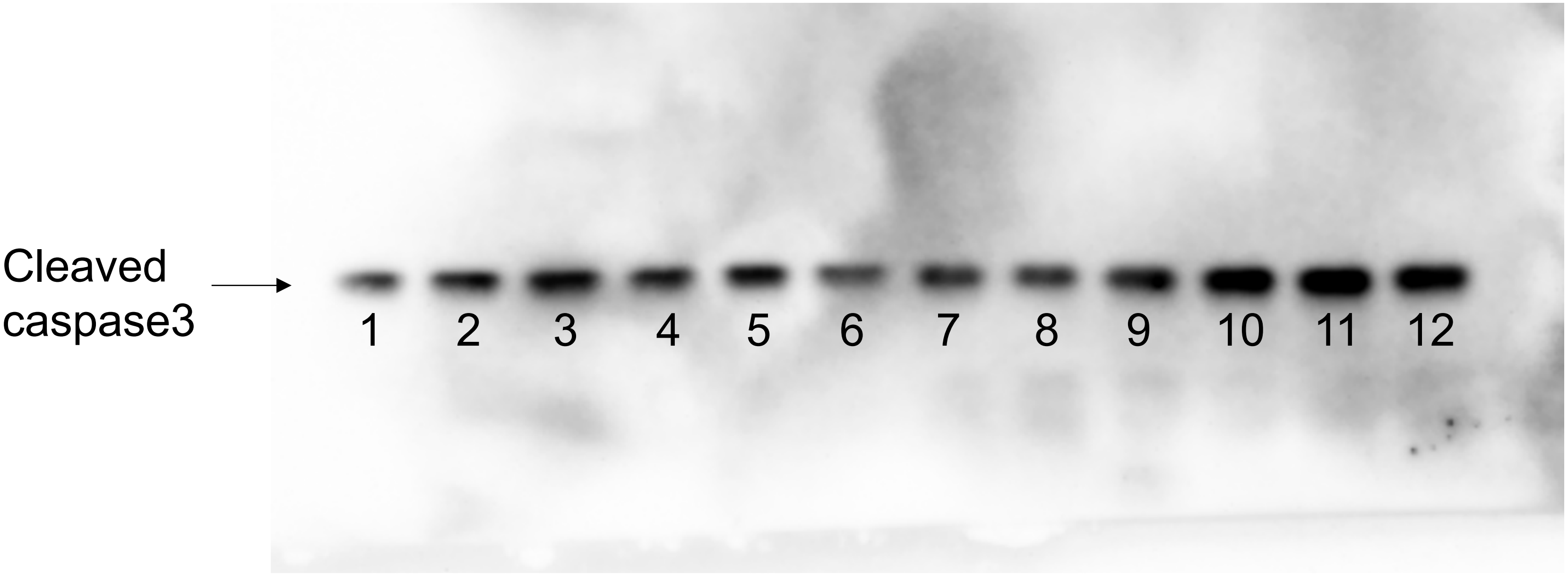

Full and uncropped western blot for Supplementary Figure 5G
Lanes 1, 2, 3, 4, 5, 6, 7, 8, 9, 10, 11, 12 are on the figure

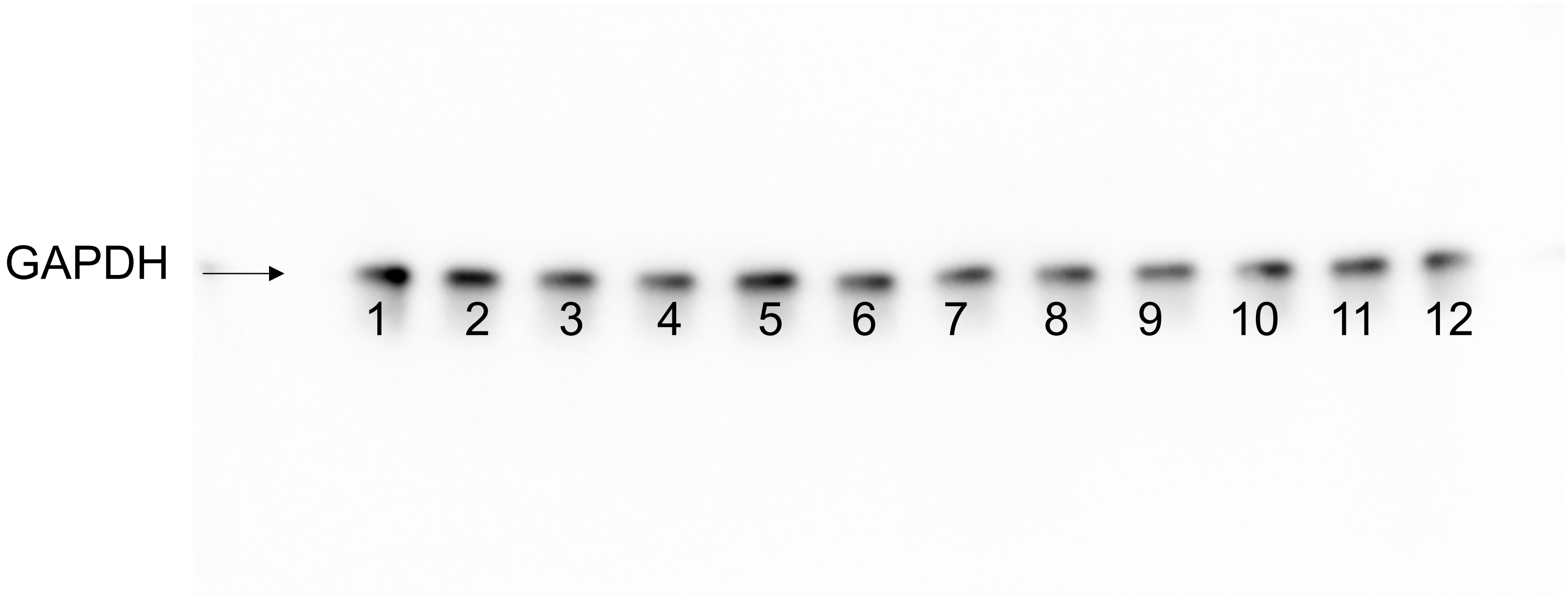

Supplement: Supplementary file 2 — Uncropped Western blot [file 41419_2024_7217_MOESM2_ESM.pdf]
